# Inactivation of Hippo pathway characterizes a poor-prognosis subtype of esophageal cancer

**DOI:** 10.1172/jci.insight.155218

**Published:** 2022-08-22

**Authors:** Zihang Mai, Jianye Yuan, Hong Yang, Shuogui Fang, Xiuying Xie, Xinye Wang, Jiaxin Xie, Jing Wen, Jianhua Fu

**Affiliations:** 1 Department of Thoracic Surgery, Sun Yat-sen University Cancer Center, Guangzhou, China.; 2 State Key Laboratory of Oncology in South China, Collaborative Innovation Center for Cancer Medicine, Guangzhou, China.; 3 Guangdong Esophageal Cancer Institute, Guangzhou, China.; 4 School of Statistics, Renmin University of China, Beijing, China.

**Keywords:** Gastroenterology, Oncology, Bioinformatics, Cancer

## Abstract

Identification of molecular subtypes that reflect different prognoses and treatment responses, especially immune checkpoint inhibitors (ICIs) in esophageal squamous cell carcinoma (ESCC), is essential for treatment decisions. We performed targeted sequencing in 201 patients with ESCC to discover genetic subtypes and validate our findings via multiple data sets. We identified 3 driver genes (*FCGBP*, *GRIN2B*, and *FRY*), and recurrent truncating mutations in *FRY* impaired its tumor-suppressive function and promoted tumor proliferation. A 3-gene mutation signature (*FAT1*, *FAT3*, and *FRY*) recognized a molecular subtype named “FAT/FRY” with frequent Hippo pathway–related mutations. In multiple ESCC cohorts, the patients with the FAT/FRY subtype had poorer prognosis than did patients in the WT group. Transcriptome analysis indicated that the FAT/FRY subtype was characterized by inactivation of the Hippo pathway, hypoxia, chemoresistance, higher infiltration of CD8^+^ T cells and activated DCs, and a transcriptome similar to that of cancer responders. Furthermore, the 3-gene signature predicted better survival for patients treated with ICIs, partially explained by its positive correlation with the tumor mutation burden and neoantigen burden. The 3-gene signature is a biomarker to recognize the FAT/FRY molecular subtype, evaluate prognosis, and select potential beneficiaries of ICIs in ESCC.

## Introduction

Although both the incidence and mortality associated with esophageal squamous cell carcinoma (ESCC) have declined due to endoscopic screening programs, ESCC remains a major cause of cancer-related death in China. To date, partially owing to our deficient understanding of genetic alterations in ESCC, the frontline therapy for advanced ESCC (*T*_1–4_*N*_+_*M*_0_) is neoadjuvant chemoradiotherapy followed by surgery (1), regardless of the tumor’s genetic background. In recent decades, some genome-wide studies have elucidated the interpatient heterogeneity of ESCC characterized by different frequently mutated genes (2, 3). Moderate coverage sequencing assays (whole-exome sequencing [WES]) often fail to detect mutations at low allelic frequencies (4) and can be complemented by targeted deep sequencing. Some studies have also sought to identify common features of heterogeneous genomes, such as mutual mutation trajectories (5, 6), that may be clinically relevant. However, mutation trajectories are still obscure in ESCC, and more investigations are needed.

Cancer heterogeneity is also reflected in genomically defined subtypes that provide biological insights into prognoses and treatment responses of patients. Molecular typing has been established in some digestive malignancies (7, 8). Recently, The Cancer Genome Atlas (TCGA) has summarized 3 ESCC molecular subtypes, but the clinical relevance of these subtypes has not been clearly elucidated (9). In parallel, the emerging immune checkpoint inhibitor (ICI) therapies have been proved to extend the survival of patients with ESCC (10), but only a minor fraction of patients benefit from ICI, stressing the importance of identifying biomarkers for predicting responses to ICIs. Some studies suggest that the tumor microenvironment and responses to immunotherapy are predetermined by the genetic basis of tumors, such as the tumor mutation burden (TMB) (11–14). However, knowledge of ESCC is limited, and there is an urgent need to explore prognostic biomarkers for molecular typing and characterize the contacts between these genetic biomarkers and immune microenvironment features, especially the responses to immunotherapy.

In this study, by applying targeted deep sequencing, we sought to discover ESCC genetic subtypes and identified a poor-prognosis subgroup of patients characterized by frequent mutations related to the Hippo pathway. Focusing on this subtype, named “FAT/FRY,” we then devised a 3-gene mutation signature to recognize it and characterized the immune profile of the FAT/FRY ESCC via multiple data sets and experimental methods. We further examined the prognostic value of the 3-gene signature in patients with cancer treated with immunotherapy. The results showed that our 3-gene signature was a promising biomarker for distinguishing an ESCC subtype with a poor prognosis for patients, who might be potential beneficiaries of immunotherapies.

## Results

### Landscape of genomic alterations.

**Figure 1:**
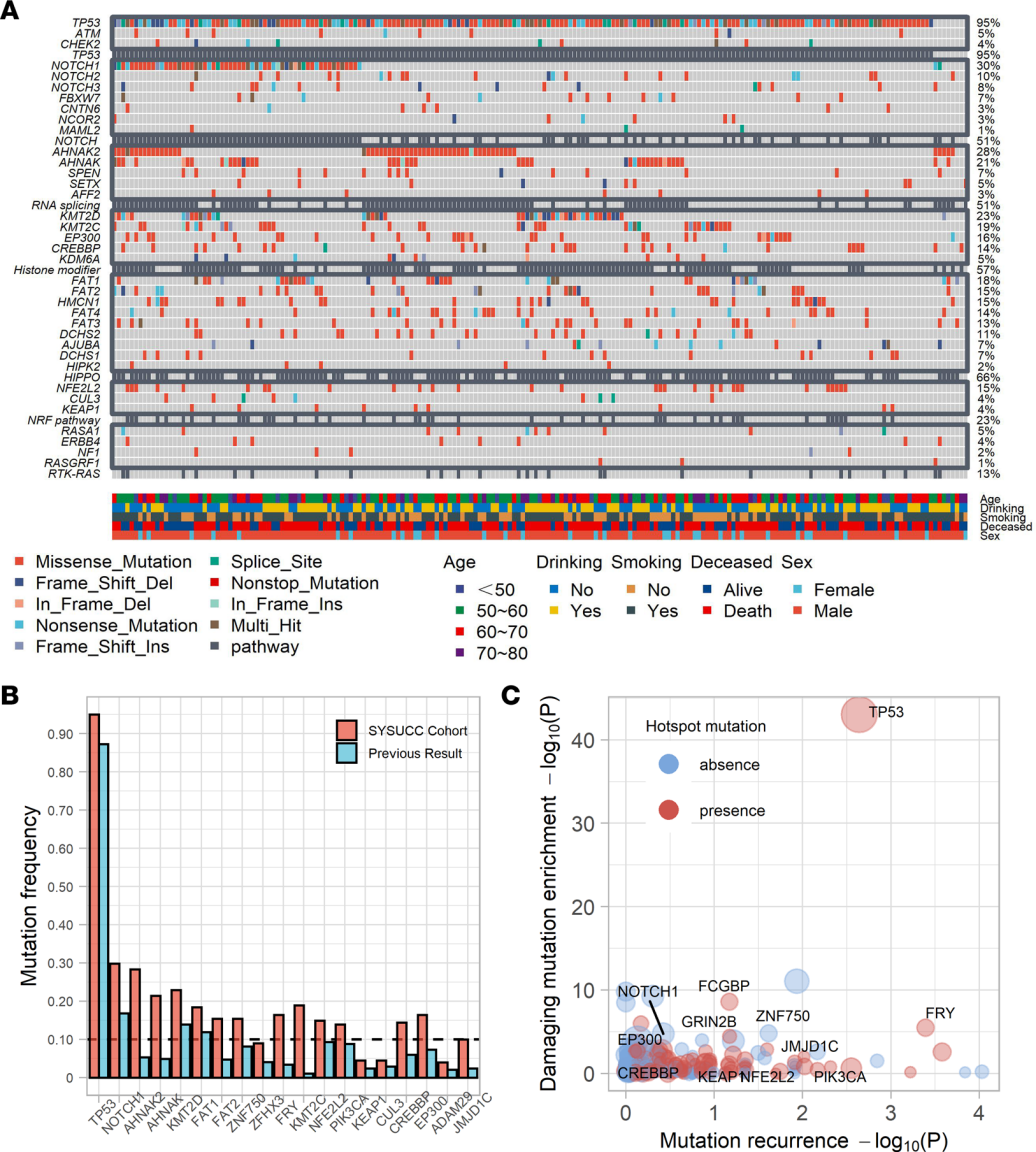
Summary of mutations in 201 patients with ESCC. (**A**) Waterfall plot displaying the mutation landscape in 201 patients with ESCC. (**B**) Histogram displaying the mutation frequencies of the cancer-associated genes observed in previous cohorts and our cohort. (**C**) Statistical assessment of mutated genes in 201 ESCC samples. Each dot represents a gene. The *x* axis and *y* axis denote the negative logarithmic transformation of *P* values from 2 statistical tests evaluating mutation recurrence (OncoClust algorithm) and the enrichment of deleterious mutations (binomial test). Del, deletion; Ins, insertion.

The genomic data of the discovery cohort (*n* = 201) were derived from our previous work (15) and were reanalyzed in this study. Our deep sequencing strategy revealed many mutations with low abundance, and the mutation frequency for most genes was higher than that observed in previous studies (2, 16–22) (Figure 1, A and B). Another 70 patients with ESCC were recruited to validate our findings. The clinicopathological parameters did not differ between both cohorts (Supplemental Table 1; supplemental material available online with this article; https://doi.org/10.1172/jci.insight.155218DS1).The analytical pipeline of this study is summarized in Supplemental Figure 2.

**Figure 2:**
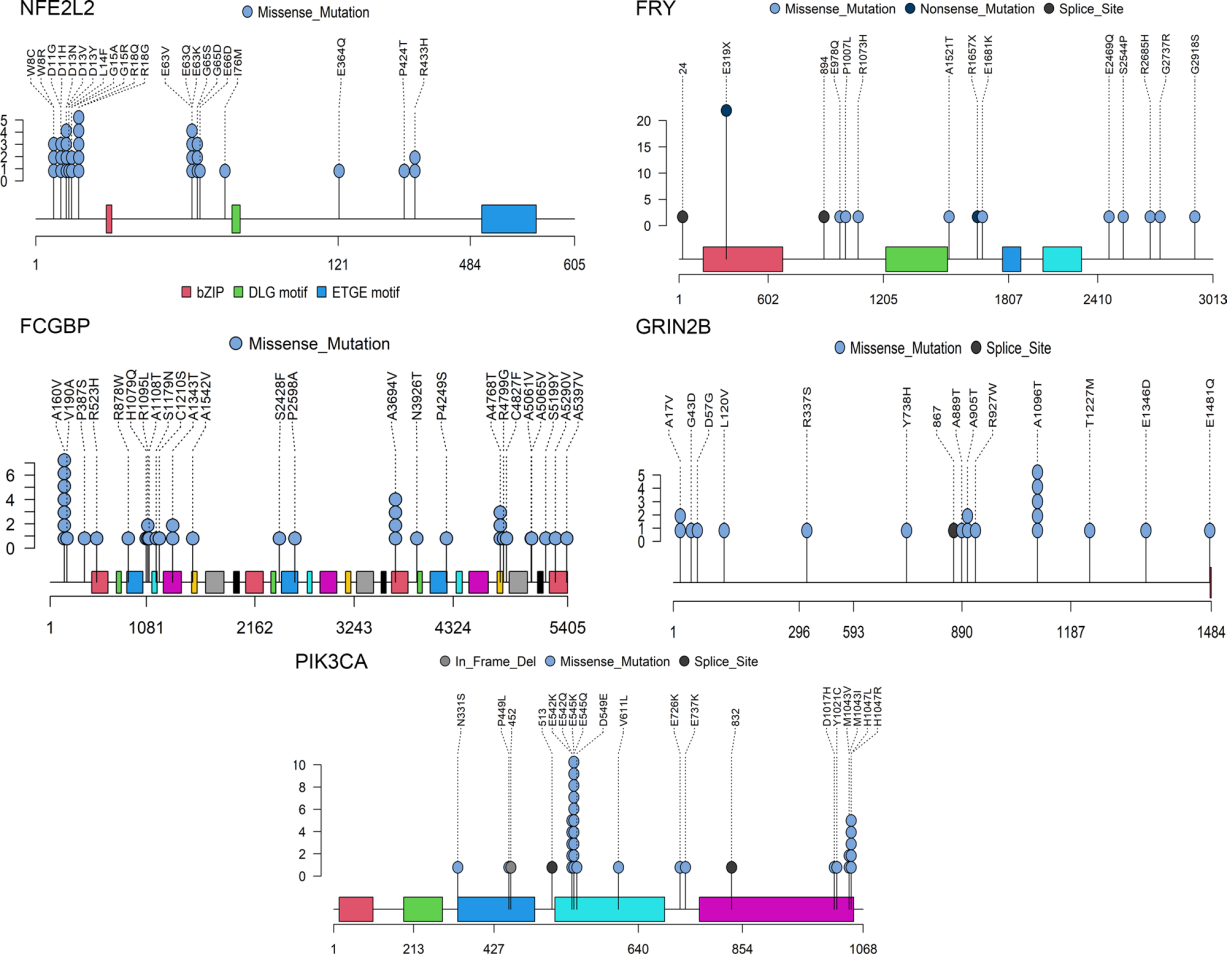
Mutation profiles of frequently mutated genes. Lollipop plot showing the mutation profiles of frequently mutated genes. Del, deletion.

**Figure 3:**
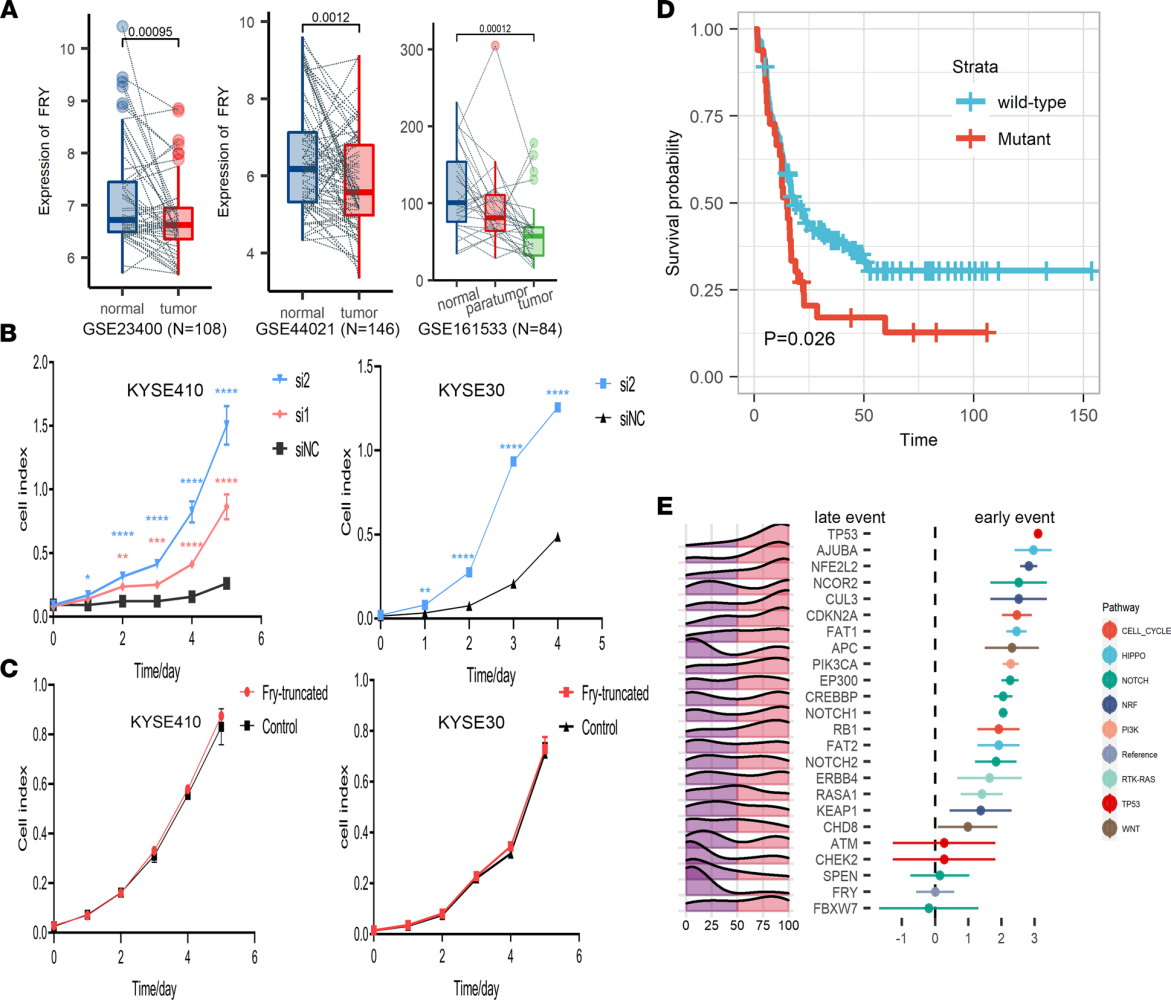
Molecular characterization of mutational driver FRY. (**A**) *FRY* mRNA levels in ESCC tumor, paratumor, and normal tissues calculated from 3 independent data sets. (**B**) Proliferation assays of KYSE410 and KYSE30 cells transfected with 2 siRNAs against FRY (si1 and si2) or control siRNA (siNC). (**C**) Proliferation assays of the FRY-truncating mutant and the corresponding control in KYSE30 and KYSE410 cells. The data in **A**–**C** represent mean ± SD; *n* = 3. *P* values were calculated by Student’s *t* test. (**D**) Survival analysis showed that the mutation status of *FRY* was significantly associated with a shorter DFS. The *P* value was calculated by log-rank test. (**E**) Inference of the relative temporal order of mutations by the Bradley-Terry model. The mountain plot in the left panel displays the distribution over cancer cell fraction of mutations in each gene. The right panel shows the temporal order of mutations, and the error bar represents the quasi-SEs.

We evaluated the recurrences, impacts of protein function, and hotspots of mutations in all mutated genes (Figure 1C) and identified 6 frequently mutated genes, *TP53* (95%), *FCGBP* (19%), *FRY* (18%), *ZNF750* (16%), *NFE2L2* (15%), and *GRIN2B* (10%) (Figure 2 and Supplemental Figure 3A), among which *TP53*, *NFE2L2*, and *ZNF750* have been proved to drive ESCC tumorigenesis (2, 3, 17). *FRY*, a key component of chromosome separation in metaphase, inhibits the growth of cancer cells in a Hippo pathway–dependent manner in some malignancies (23, 24). Analysis of 3 public microarray data sets revealed that the *FRY* mRNA expression was significantly downregulated in ESCC compared with matched normal tissues as well as paratumor tissues (Figure 3A). Analysis of public data sets demonstrated the expression of FRY also inversely correlated with the expression of YAP at both mRNA and protein levels (Supplemental Figure 4, A and B), which was the main effector of the Hippo pathway. FRY was also negatively associated with many downstream targets of Hippo/YAP (Supplemental Figure 4). We further measured the transcriptional targets of Hippo/YAP after silencing FRY and found an increase of both CTGF and CYR61 in knockdown cells as compared with the control (Supplemental Figure 3D), supporting the role of FRY as an inhibitor of Hippo/YAP (23, 24). A total of 33 samples contained *FRY* mutations, among which 25 were loss-of-function mutations, including *FRY* p.E319X.

The functions of *FRY* and its hotspot mutations in ESCC remain to be determined. In 2 ESCC cell lines with WT endogenous *FRY* expression (Supplemental Figure 3B), we independently silenced *FRY* with siRNA and found that the depletion of *FRY* significantly promoted cell proliferation (Figure 3B). Because of the low expression of FRY in KYSE30 cell line, it was hard to further knock down its expression, so we only successfully performed RNAi to knock down FRY with 1 siRNA (Supplemental Figure 3E). Ectopic expression of the *FRY* hotspot mutant p.E319X did not mimic the WT *FRY* and had no effects on cell proliferation, supporting the loss-of-function role of *FRY* p.E319X (Figure 3C and Supplemental Figure 3F). Among the 6 frequently mutated genes, *FRY* was the only gene associated with shorter disease-free survival (DFS), and this association remained significant after adjusting for clinicopathological factors (adjusted HR, 1.61 [95% CI, 1.06–2.44]; *P* = 0.026) (Figure 3D and Supplemental Figure 3C). These analyses suggested that recurrent inactivation of FRY promoted ESCC progression and disease relapse.

At codon resolution, we detected 133 mutational hotspots, which were deemed functional events, because hotspot mutations can accumulate under positive environmental selection (25) (Supplemental Table 2). Consistent with a previous report (21), we also observed the accumulation of hotspot mutations in histone modifiers, supporting their oncogenic roles in ESCC (Supplemental Figure 3A). The mutation profile of *PIK3CA* was dissimilar to that of breast cancer, in which hotspot mutations in the helical domain (p.E542, p.E545) were more common than PI3K/PI4K (p.E1047) hotspot mutations (26) (Figure 2). We also detected the hotspot mutations p.R18 (*n* = 5) and p.E63 (*n* = 4) in the *KEAP1*-binding domain of *NFE2L2*, and these hotspot mutations might activate *NFE2L2* by disrupting its interaction with *KEAP1*.

### Mutation trajectory of ESCC.

Throughout tumor progression, oncogenic mutations in cancer-associated genes promote tumor growth and thus propel clonal expansion. The phylogenetic relationship of mutations has been documented (27), but the limited sample size and sequencing depth prevent the elucidation of the common evolutionary trajectories of oncogenic mutations.

To address this issue, we first identified oncogenic mutations through a literature review (Supplemental Methods). We then applied the Bradley-Terry model to obtain statistical evidence of temporal orders (6) (Supplemental Table 3). We observed that the progression of ESCC followed a mutation trajectory at the pathway level (Figure 3E). TP53 was an early mutation target in ESCC, similar to observations of other cancer types (5). Mutations affecting the *NRF* pathway, including *NFE2L2* and its degrading element *CUL3*, were also acquired early. *NOTCH* pathway–related genes were mutated continually during clone expansion, whereas *FRY* mutations were acquired later. Theoretically, early events tended to occur in all tumor cells, and later events were carried by minor clones. Then, we calculated the cancer cell fractions of mutations and inferred their clonal statuses (Supplemental Table 4). Oncogenic mutations in *TP53* (FDR < 10^–6^) and *NFE2L2* (FDR = 0.009) tended to be clonal, and all the oncogenic mutations in *FRY* (FDR = 0.01) were subclonal, verifying the temporal order of these events.

### Prognostically distinct subtypes of ESCC.

**Figure 4:**
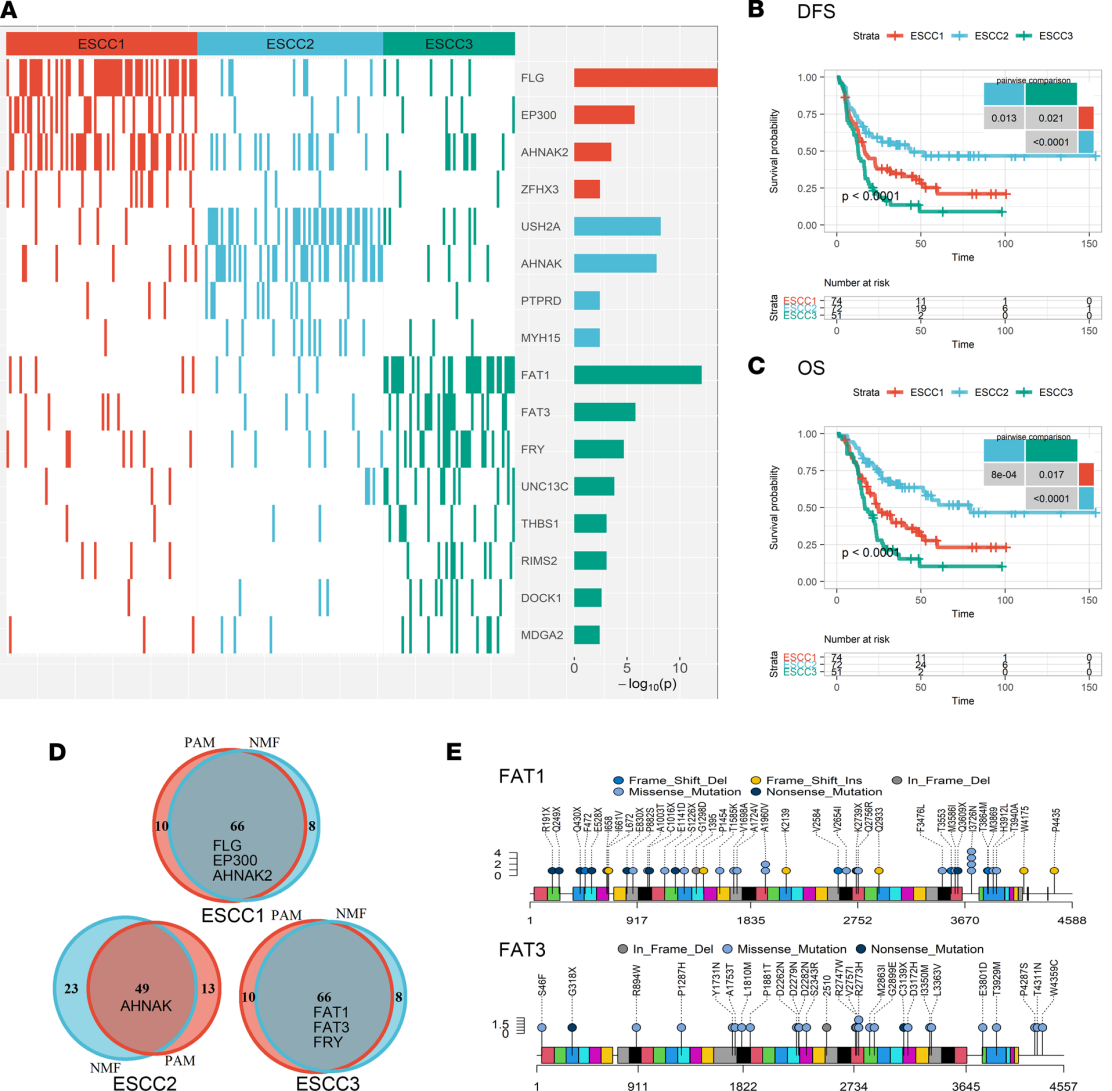
Molecular subtypes of ESCC. (**A**) Heatmap of characteristic mutations from 3 clusters. Distinct clusters are labeled by colors. The histogram on the right-hand side shows the enrichment of mutations within clusters on a negative logarithmic *P* value scale. Only mutations identified as significantly enriched in the given group, as determined by a Benjamini-Hochberg–adjusted FDR ≤ 0.2 according to Fisher’s exact test, are displayed. (**B** and **C**) Three clusters of patients had distinct DFS (**B**) and OS (**C**) rates. *P* values of global and pairwise comparisons were generated by log-rank tests. (**D**) Venn plot displaying the significant overlap of clusters identified by 2 independent algorithms. Mutual marker mutated genes of clusters identified by 2 algorithms were placed in the overlapping field. (**E**) Lollipop plot displaying the mutation distributions of *FAT1* and *FAT3*. Del, deletion; Ins, insertion; PAM, partitioning around medoids consensus clustering.

Theoretically, the dissimilar prognoses of patients with ESCC could be explained by heterogeneous patterns of prognosis-associated mutations. According to the distribution of prognosis-associated mutations, we identified 3 clusters of patients by applying nonnegative matrix factorization (NMF) consensus clustering (28). A separate algorithm based on partitioning around medoids consensus clustering (29) reproduced significantly overlapping clusters (Figure 4D; kappa index: 0.734; *P* < 10^–14^). The consistent results obtained by 2 distinct algorithms suggested the clusters were robust. We termed the clusters from NMF ESCC1, ESCC2, and ESCC3, then compared mutation frequencies of all the genes in the 3 clusters using Fisher’s exact test to define characteristic mutations for each cluster (Figure 4A).

ESCC1 had higher rates of mutations in *FLG* (62%; FDR < 10^–10^), *AHNAK2* (45%; FDR = 0.029), and *EP300* (34%; FDR < 0.001). *EP300* is a putative driver of NOTCH pathway that is frequently mutated in ESCC and indicates an adverse prognosis (21, 25). *AHNAK2* has a high mutation frequency in ESCC, and its oncogenic role has been reported in multiple malignancies (30). *USH2A* (mutation rate, 46%; FDR < 10^–5^) and *AHNAK* (mutation rate, 44%; FDR < 10^–5^) mutations surrogated ESCC2, and patients in the ESCC2 cluster had the best prognosis among those in all 3 clusters (Figure 4, B and C). *USH2A* is frequently mutated in tongue squamous cancer (31), and *AHNAK* is associated with tumor metastasis (32). Interestingly, mutations in *AHNAK* and *AHNAK2*, both of which are involved in RNA splicing, represented clusters with dissimilar prognoses, implying their different roles in ESCC progression.

ESCC3 was dominated by mutations of *FAT1* (mutation rate, 55%; FDR < 10^–9^), *FAT3* (mutation rate, 25%; FDR < 0.001), and *FRY* (mutation rate, 37%; FDR < 0.003), which were major regulators of the Hippo pathway. *FAT1*, a tumor suppressor recurrently inactivated in ESCC (2), could dampen the activity of the Hippo effector *YAP* (33, 34). Here, we detected 33 *FAT1* mutations in ESCC3, including 16 truncating and 6 missense mutations recorded in the Catalog of Somatic Mutations in Cancer (35) (Figure 4E). ESCC3 also had a high mutation frequency of *FAT3*, which is the most homologous protein to *FAT1* in the *FAT* family (36). Another frequently mutated gene in ESCC3 was *FRY*, which was proved to inhibit *YAP* by blocking its nuclear transportation (23, 24). Loss-of-function mutations in *FRY* were observed in 29.4% (*n* = 15 of 51) of patients in the ESCC3 cluster, as we mentioned above (Figure 2). Together, ESCC3 was characterized by the mutations of *FAT1*, *FAT3*, and *FRY*, which might lead to the inactivation of the Hippo pathway. Notably, ESCC3 had the worst DFS and overall survival (OS) among all 3 clusters (Figure 4, B and C).

### A 3-gene mutation signature for prognosis prediction.

**Figure 5:**
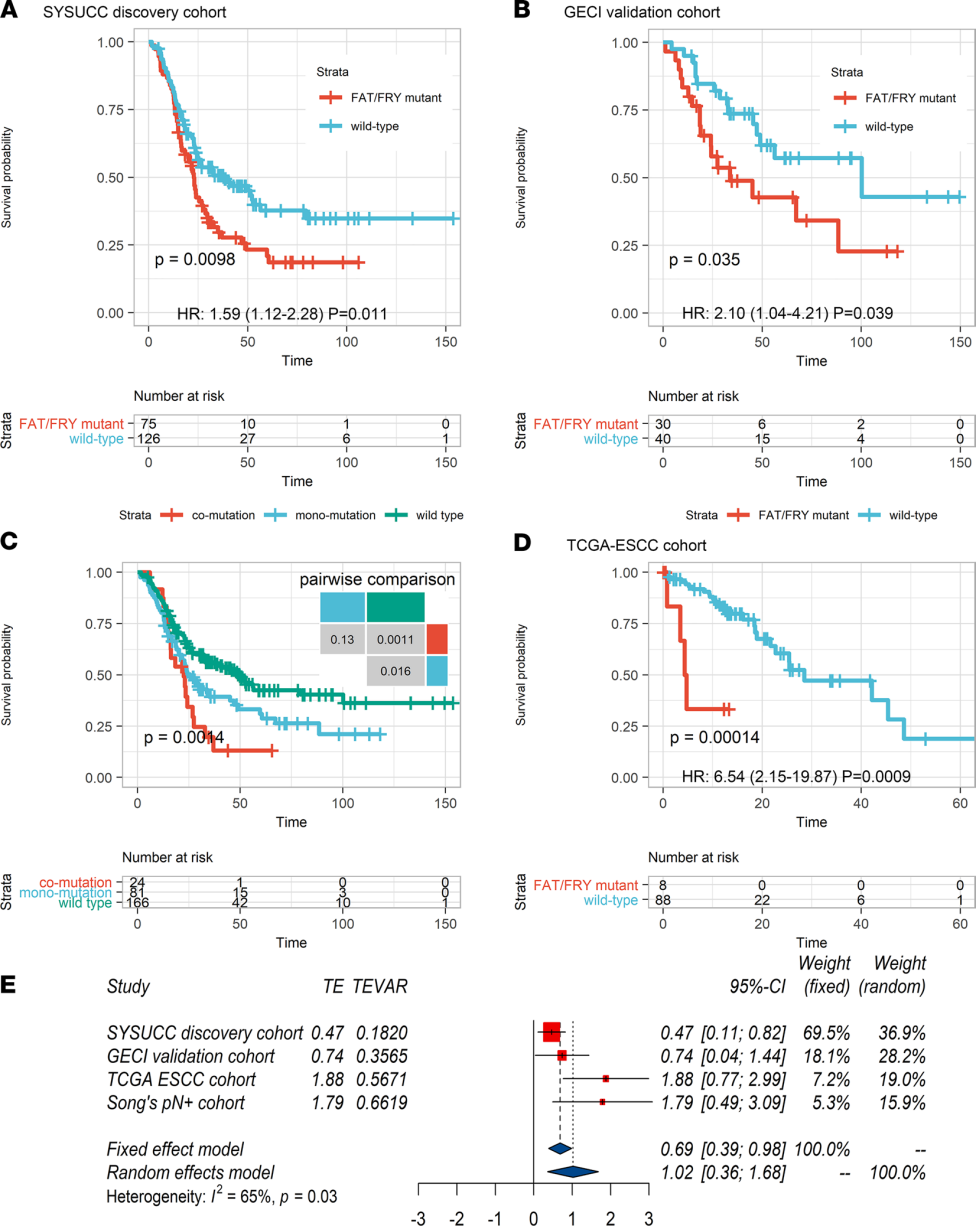
Identification of a 3-gene signature associated with shorter OS for patients with ESCC. (**A** and **B**) Kaplan-Meier survival analysis showed that patients with mutation(s) in at least 1 gene of this signature had significantly shorter OS than patients with WT genes in our discovery (**A**) and validation cohorts (**B**). (**C**) Survival curves show a marginal trend in which patients with comutations had worse OS than patients with 1 mutated gene in the 3-gene signature. (**D**) The FAT/FRY subgroup had worse survival in TCGA-ESCC cohort (*n* = 96). *P* values in **A**–**D** were calculated by log-rank tests. (**E**) Forest plot of the HRs of death in different molecular subgroups from 4 cohorts with the random effects model (pooled HR, 2.77 [95% CI, 1.43–5.36]; *I*^2^ = 65%; *P* = 0.03, *I*^2^ test). TE, target estimate; GECI, Guangdong Esophageal Cancer Institute; Song’s pN+ cohort, ref. 20.

Based on the molecular features enriched in ESCC3, we developed a signature consisting of *FAT1*, *FAT3*, and *FRY* to recognize this subtype. Patients with 1 or more mutation(s) in these genes were assigned to this subtype, which was termed FAT/FRY. Patients in the FAT/FRY subgroup had shortened OS and DFS (Figure 5A and Supplemental Figure 5A) compared with those in the WT subgroup independent of the TNM stage and other clinicopathological factors (Supplemental Table 5). The median OS duration of patients in the FAT/FRY subgroup was 22.8 months compared with 39.6 months in the WT subgroup (HR, 1.59 [95% CI, 1.12–2.28]; *P* = 0.011, Cox regression). The independent prognostic value of the 3-gene signature was also confirmed in our validation cohort (HR, 2.1 [95% CI,1.04–4.21]; *P* = 0.039) (Figure 5B and Supplemental Figure 5B).

Because *FAT3* was frequently comutated with *FRY* (OR, 3.10; *P* = 0.021, Fisher’s exact test) and *FAT1* (OR, 2.59; *P* = 0.057), we found a trend of dosage effects indicating patients with comutations had a worse DFS (*P* = 0.052, log-rank test) and OS (*P* = 0.13) than those with 1 mutated gene (Figure 5C and Supplemental Figure 5C). This trend could be explained by the functional redundancy of *FAT1* and *FAT3* (34) and the inactivation of *YAP* by *FRY* in a Hippo pathway–independent manner (23). We also observed that patients in the FAT/FRY subgroup in the N1 stage had a similar prognosis (*P* = 0.47) as patients in the WT subgroup in the N2 stage (Supplemental Figure 6F). Furthermore, the 3-gene mutation signature was not confounded by the TNM stage or other clinicopathological parameters (Supplemental Table 6). Finally, we compared the predictive capacity of the 3-gene signature with that of the TNM stage. The area under the curve of the 3-gene signature was similar to that of the TNM stage (*P* > 0.5) (Supplemental Figure 7).

We further verified this prognostic biomarker in independent data sets (9, 20). In TCGA-ESCC cohort, the survival of patients in the FAT/FRY subgroup was significantly shorter than that of patients in the WT subgroup (HR, 6.54 [95% CI, 2.15–19.87]; *P* = 0.0009, Cox regression) (Figure 5D and Supplemental Figure 5D), and a similar significance was observed in another ESCC data set (Supplemental Figure 5G). Then, we pooled the HR from 4 cohorts using a random-effect model and found that the risk of cancer-related death in the FAT/FRY subgroup was augmented by 177% compared with the WT subgroup, despite the moderate heterogeneity (pooled HR, 2.77 [95% CI, 1.43–5.36]; *I*^2^ = 65%; *P* = 0.03).

### Molecular characteristics of the FAT/FRY subtype.

We further compared the frequencies of mutations between 2 molecular subgroups. As shown in Supplemental Table 9, we found that mutations of 3 genes were the dominant features in the FAT/FRY subtype, and we did not observe enrichments of mutations in oncogenic pathways like NRF2 and NOTCH, implying that mutations of other pathways would not provide selective advantages or lead to synthetic lethality (25). On the other hand, some ESCC-associated genes were frequently mutated in the WT group, including RB1, FAM135B, and NOTCH3 (*P* < 0.05, Fisher’s exact test) (19, 20).

**Figure 6:**
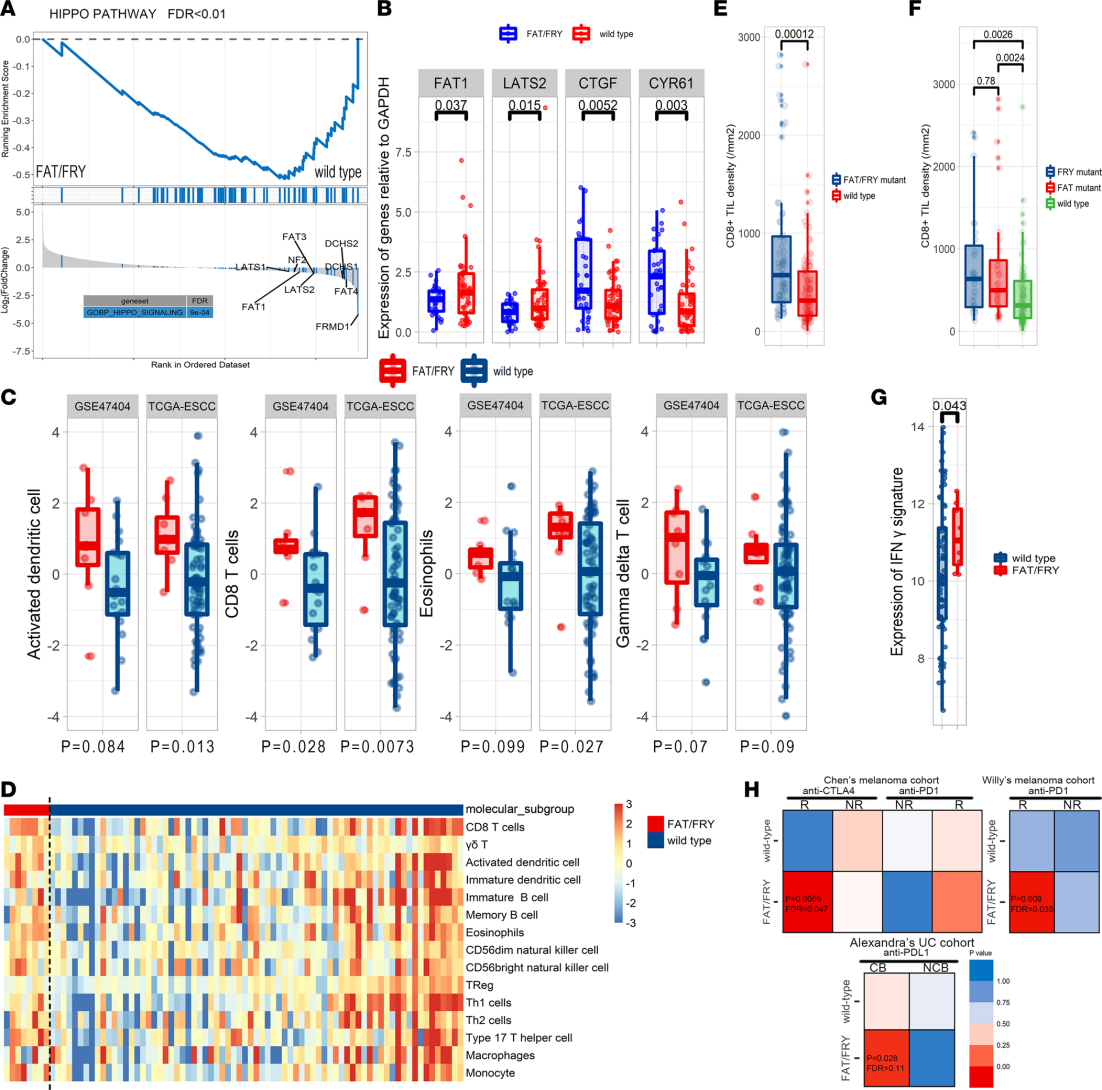
Molecular and immune microenvironment characteristics of FAT/FRY subtype ESCC tumors. (**A**) Gene set enrichment analysis plot showing that genes involved in the Hippo pathway were dysregulated in the FAT/FRY subgroup. (**B**) The expression level of core genes of the Hippo pathway and targets of Hippo/YAP in our discovery set (*n* = 90). (**C**) Box plot showing the differences in the relative abundance of CD8^+^ T cells, activated DCs, eosinophils, and γδ T cells between both groups in the TCGA-ESCC and GSE47404 data sets. (**D**) Heatmap displaying the relative abundances of major immune cell types in TCGA cohort. (**E** and **F**) Validation of enrichment of CD8^+^ TILs by IHC in our discovery cohort (*n* = 170). (**G**) Box plot showing the differences in the expression of IFN-γ response signature between the FAT/FRY and WT groups. (**H**) Transcriptomic similarities of our ESCC subtypes and response groups in 3 ICI cohorts: Pei-Ling Chen et al. (55), Willy Hugo et al. (42), and Alexandra Snyder et al. (56). A smaller *P* value indicates a higher transcriptomic similarity between the 2 groups. The *P* values in **B**–**G** were calculated by Wilcoxon test. The box plots depict the minimum and maximum values (whiskers), the upper and lower quartiles, and the median. The length of the box represents the interquartile range. NR, nonresponse; R, response.

Taking advantage of the multiomics data from TCGA, we depicted the transcriptomic features of the 2 ESCC subgroups. The gene set enrichment analysis showed that Hippo pathway–related genes were significantly enriched in downregulated genes in FAT/FRY–subtype tumors, confirming the inactivation of the Hippo pathway at the transcriptional level (FDR = 0.006) (Figure 6A). In our discovery cohort, we observed that the core members (FAT1 and LATS2) of Hippo pathway were significantly underexpressed in the FAT/FRY ESCC subtype, whereas the transcriptional targets of YAP1 (CTGF and CYR61) were significantly increased, verifying the inactivation of Hippo pathway in this subgroup (Figure 6B). The gene sets of FAT/FRY samples were enriched in hypoxia- and drug metabolism–related pathways (*P* < 0.05; FDR < 0.25) (Supplemental Figure 11, A and B).

We further asked if the hypoxia and drug metabolism background was responsible for the poor prognosis of patients in the FAT/FRY subgroup. As shown in Supplemental Figure 11, C and D, patients with a higher score for hypoxia- and drug metabolism–related pathways had worse outcomes, so the enhanced aggressiveness of tumors in the FAT/FRY subgroup might be induced by overactivation of YAP1-, hypoxia-, and drug metabolism–related pathways.

Increasing evidence has demonstrated the essential roles of the Hippo pathway in the regulation of immunity (33, 37), prompting us to investigate the association between the 3-gene signature and the tumor immune microenvironment. We found that the FAT/FRY subgroup had higher infiltration of CD8^+^ T cells, activated DCs and eosinophils, and slightly higher levels of γδ T cells (Figure 6, C and D). This phenomenon was replicated in another ESCC multiomics data set (Figure 6C). We also performed IHC staining on whole-tumor slides of our discovery cohort to quantify densities of CD8^+^ tumor-infiltrated lymphocytes (TILs), the main factor in antitumor immunity and an indicator of immunotherapy efficacy (38). Accordingly, we found an augmented infiltration of CD8^+^ TILs in FAT/FRY subtype ESCC, and tumors with FRY mutant alone also had more CD8^+^ TILs compared with WT tumors, suggesting that FAT/FRY tumors still had active antitumor immunity (Figure 6, E and F).

### Molecular subtype–based treatment strategy for ESCC.

Because nearly half of the patients in our study received adjuvant therapy (ADT) that was believed to improve prognoses (Supplemental Figure 10, A and B), we further evaluated, using the Cox model, whether our FAT/FRY signature was a predictive biomarker of ADT efficacy or prognosis biomarker (39). An insignificant treatment-by-biomarker interaction indicated that the prognostic value of FAT/FRY signature did not differ between 2 treatment groups (OS, *P* = 0.8; DFS, *P* = 0.6) (Supplemental Figure 10, C–F). Moreover, we confirmed the prognostic value of the FAT/FRY signature in a subgroup of patients without ADT (Supplemental Figure 10, G and H), signifying its role as a prognostic biomarker.

To further determine whether patients with ESCC in the FAT/FRY subgroup were potential beneficiaries of ICIs, we first evaluated the expression of an IFN-γ signature, which was previously reported to correlate with a better response to ICIs (40). The FAT/FRY subgroup had significantly higher expression of the *IFN-γ* signature in the TCGA-ESCC data set (Figure 6G). Notably, 2 of 6 genes in this signature were not detected in GSE47404 and were not adequate for signature validation. We next applied the subclass mapping (SubMap) algorithm to globally assess the similarity of transcriptome profiles of patients in FAT/FRY subgroups with those of patients in 2 melanoma cohorts and 1 urothelial cancer cohort with distinct responses treated with ICIs (41, 42). A lower *P* value yielded from SubMap represented a higher similarity between the 2 groups. From the transcriptomic perspective, FAT/FRY ESCC subtypes were similar to the responders in all 3 ICI cohorts (*P* < 0.05) (Figure 6H), indicating potential immunotherapy benefits for FAT/FRY ESCC.

**Figure 7:**
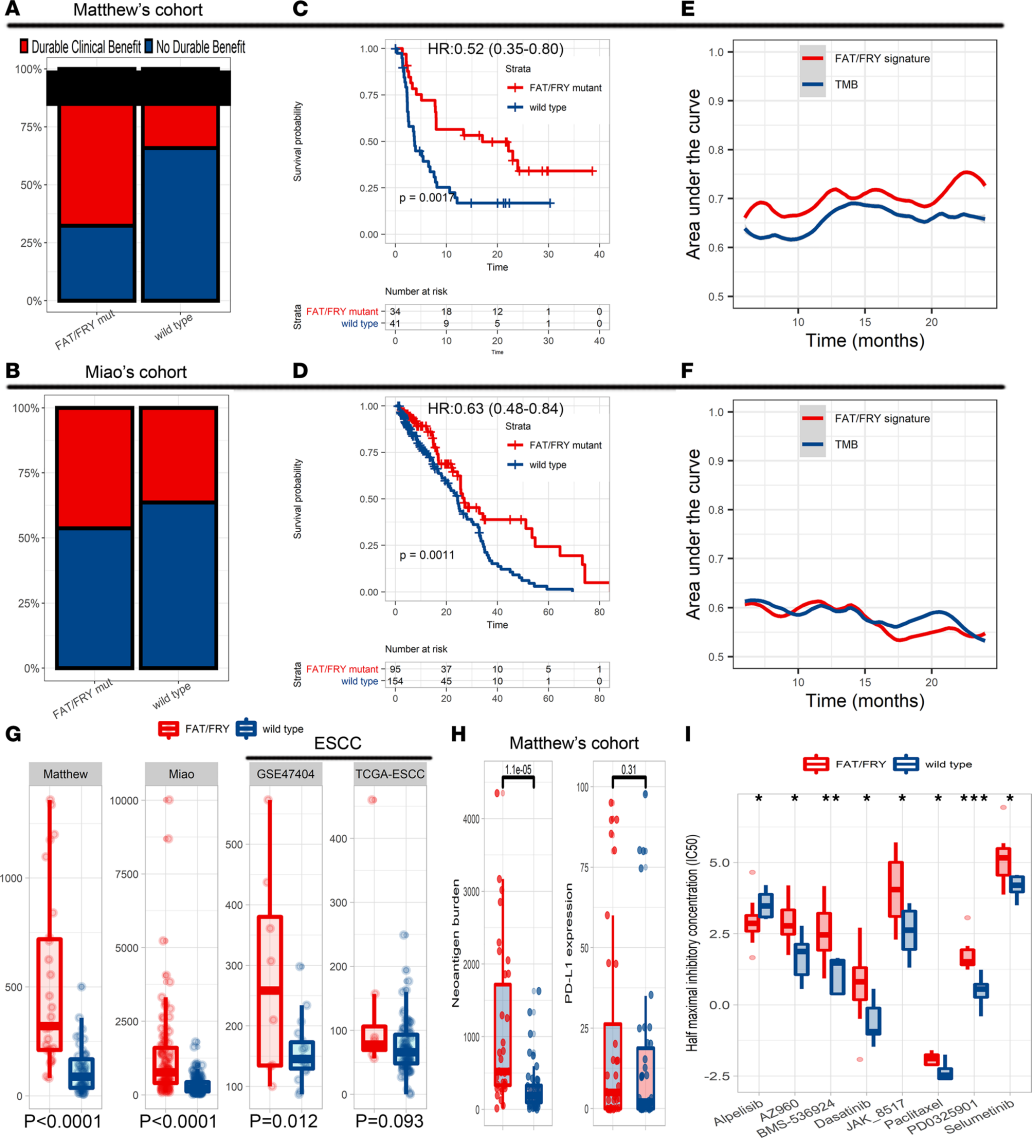
Molecular subtype–based treatment strategy. (**A** and **B**) Bar chart showing that the FAT/FRY subgroup had higher rates of durable clinical benefit than the WT group in both ICI data sets. (**C** and **D**) Survival differences of the FAT/FRY subgroup and WT subgroup in Matthew Hellmann’s (13) and Diana Miao’s (12) ICI cohorts. *P* values were calculated by log-rank tests. (**E** and **F**) The area under the time-dependent received operator characteristic curve of the 3-gene signature and TMB to predict post-ICI prognosis in the cohorts from refs. 12 and 13. (**G**) Box plot indicating that the significant correlations of the 3-gene signature and TMB in 2 ICI and 2 ESCC cohorts. (**H**) Box plot showing that the FAT/FRY subgroup had higher neoantigen burden but not *PD-L1* expression than the WT subgroup. (**I**) Box plot displaying differences of drug responses in FAT/FRY-mutant ESCC cell lines and WT cell lines in the GDSC pharmacogenomics database. *P < 0.05, **P < 0.01, ***P < 0.001. *P* values in **G**–**I** were calculated by Wilcoxon test. The box plots depict the minimum and maximum values (whiskers), the upper and lower quartiles, and the median. The length of the box represents the interquartile range.

Moreover, we assessed the prognostic value of the 3-gene signature in patients with immunotherapies. Because of the lack of mature sequencing data on ESCC, we chose a cohort with microsatellite stable tumors and a non–small cell lung cancer (NSCLC) cohort to validate its predictive capacity (11–13) because of the prevalence of microsatellite stable status in ESCC and a similar genomic background of NSCLC to ESCC (9). As expected, the signature significantly correlated with higher rates of durable clinical benefit (Figure 7, A and B). As shown in Figure 7, C and D, the FAT/FRY subgroup had longer progression-free survival and OS than the WT group in 2 ICI cohorts. Compared with high TMB (TMB-H), the signature achieved a more significant stratification in selecting potential responders of ICIs (log-rank *P* value, 0.0017 < 0.0061 in Matthew Hellmann et al.’s cohort, ref. 13; and 0.0011 < 0.0075 in Diana Miao et al.’s cohort, ref. 12) (Supplemental Figure 11, E and F). In Hellmann et al.’s cohort (13), the AUC of our 3-gene signature was higher than that of the TMB across different time points (Figure 7E), whereas in Miao et al.’s cohort (12), the prediction performance of the signature was higher at 12-month follow-up but relatively worse in the 18-month follow-up compared with the performance of TMB (Figure 7F). Therefore, we suggested that the predictive capacity of our 3-gene signature was comparable to that of WES-based TMB in both ICI cohorts. In another ICI cohort, we could not fully validate the prognostic value of the signature because of insufficient coverage of the Memorial Sloan Kettering Cancer Center panel, but we observed a significant correlation of FAT1 mutation with longer OS (Supplemental Figure 11G). Behind this phenomenon, we observed that the 3-gene mutation signature was positively correlated with the TMB in both the ESCC and 2 ICI data sets (Figure 7G). In Hellmann et al.’s data sets, we also found higher neoantigen burden instead of *PD-L1* expression in the FAT/FRY subgroup tumors (Figure 7H), which might drive active antitumor immunity. We could not validate this association in other data sets because of the lack of data.

Furthermore, the public pharmacogenomics database Genomics of Drug Sensitivity in Cancer (GDSC) was used to screen the responses of the molecular subtypes to common chemotherapeutic agents. As shown in Figure 7I, the FAT/FRY-mutant ESCC cell lines were more resistant to multiple agents, including paclitaxel and dasatinib, but more sensitive to alpelisib (*P* < 0.05), stressing the potential application of alpelisib in the FAT/FRY ESCC subtype.

Additional study data may be found in Supplemental Tables 7 and 10 and Supplemental Figures 5, E and F; 8; and 9.

## Discussion

Advanced ESCC is a genetically and clinically heterogeneous disease, but treatments for patients with ESCC are guided by the TNM system empirically, regardless of the tumors’ genetic basis. Although several genome-wide studies have been conducted, useful biomarkers for prognosis assessment and treatment decision are still limited (2, 3, 21). Our study covered the most frequently mutated gene loci across the ESCC genome for the in-depth genomic characterization of a large cohort, enabling us to associate genetic features with clinical characteristics.

According to evolutionary theory, activating mutations in oncogenes tend to cluster within specific regions, and most mutations are deleterious (17). Following this rule, we found that in addition to other known driver genes, mutations in *FRY*, *FCGBP*, and *GRIN2B* might exert oncogenic effects on ESCC progression. Among these genes, the mutation status of *FRY* was associated with rapid disease relapse. We also performed a set of functional assays to confirm that truncating mutations in *FRY* might dampen its effect as a tumor suppressor. Taken together, these results showed that *FRY* mutations might serve as delayed driver events conferring tumor relapse.

Delineating the temporal order of oncogenic mutations is important for identifying therapeutic targets, because targeting early clonal events can affect entire ESCC cell populations. For example, *PIK3CA* mutations are intermediate events and are commonly found in tumor subclones (21 of 29 mutations are subclonal), which may account for the variable responses to emerging PIK3CA therapies (26). Mutations in the NRF pathway genes, including *NFE2L2*, the indicator of poor prognosis, and its degrading element *CUL3*, are early events in ESCC (3). Taken together, these results show that mutations in the *NRF* pathway occur early in disease development and determine patients’ poor prognosis, offering helpful information for early detection of ESCC (3). Note that our conclusions are based on observations from a retrospective cohort and further validations require prospective studies of successive samples.

Unsupervised clustering analysis of prognosis-associated mutations led to the identification of 3 robust patient subpopulations with distinct prognoses. Notably, the group with the worst prognosis showed severe mutational lesions in the Hippo pathway (frequent *FAT1*, *FAT3*, and *FR*Y mutations). Coincidentally, a multiomics study based on TCGA also reported an ESCC cluster characterized by Hippo pathway alterations (i.e., *YAP* amplification and *VGLL4/ATG7* deletion) (9).

A clinically relevant finding of our study is the development and verification of a 3-gene mutation signature to predict patient survival as well as to select potential responders to immunotherapy. Patients with this mutation signature had a graver prognosis than those with WT alleles, indicating the potential of this signature to help distinguish a molecular subtype with poor prognosis. Having a hypoxia and chemoresistance transcriptome background, the FAT/FRY tumors still had higher immune infiltrates, especially for CD8^+^ TILs. Note that the poor-prognosis and higher CD8^+^ TIL infiltrations in FAT/FRY-mutant ESCC were not contradictory, because CD8^+^ TILs was not a robust indicator of survival in this less immunogenic tumor type (43). A meta-analysis revealed the insignificant associations between CD3^+^CD4^+^ T cells and patient prognosis, as well as significant heterogeneity in correlations between CD8^+^ TILs and prolonged survival; insignificant results were reported in more than a half of the studies (*n* = 7 of 13) (44). Our results might provide insights into genetic determinants of CD8^+^ TILs’ effects on patient prognosis, showing that overactivation of the strong oncogene YAP1 might abate the effects of immune infiltrations and lead to poor prognosis.

Compared with the prognostic predictors that were used to calculate the risk scores for patients based on the expression (45) or mutation status (46) of biologically irrelevant genes, our prognostic signature, defined by a simple rule, is more practical to identify in the clinic patients with a poor outcome, and these patients should receive more radical treatments.

The emerging ICIs have been widely used in clinical work because of their durable benefit. However, the unaffordable expense, low response rates, and the lack of response biomarkers make them less cost-effective in patients with ESCC. In our study, several genetic clues implied that this ESCC subgroup might respond to ICIs: (1) higher TMB; (2) active infiltrations of immune cells, especially CD8^+^ T cells, which were predictive of ICIs; (3) higher expression of an IFN-γ–related signature, which was confirmed as a predictor of responses to pembrolizumab in KEYNOTE-012 and KEYNOTE-028 (40); and (4) high similarity of the FAT/FRY ESCC expression profile to those of ICI responders. Because of the lack of available ICI data sets in ESCC, we instead examined the prognostic value of our 3-gene signature in cohorts of other cancer types that were genetically similar to ESCC. The promising results demonstrated that the 3-gene mutation signature could differentiate both short-term (clinical benefit or not) and long-term survival (progression-free survival or OS) outcomes in patients treated with ICIs. In our study, the performance of the signature was comparable to that of TMB, an FDA-approved pan-cancer biomarker for ICIs. Moreover, our mutation signature consists of only 3 genes, and thus it is easier to detect and more cost-efficient than TMB based on WES or panel sequencing. In the future, the maturity of our prospective trial (ClinicalTrials.gov identifier NCT04006041) on discovering biomarkers of ICI in ESCC would provide direct evidence of the prediction capacity of our signature.

Additionally, drug-response analysis from the GDSC database hinted that FAT/FRY subtype ESCC cell lines might be more sensitive to the PIK3Ca inhibitor alpelisib. This drug reduces tumor growth via inhibiting the phosphorylation of PI3K downstream targets (47), such as Akt. In FAT/FRY tumors, inactivation of Hippo pathways leads to overactivation of YAP1, the main consequence of which is the hyperactivity of the PI3K/Akt pathway (48, 49). Alpelisib can also abate effects of YAP-PI3K-Akt and inhibit tumor growth. Complex crosstalk between the PI3K/Akt pathway and other pathways, like the Hippo pathway, might also affect drug efficacy (48, 49). More efforts are needed to determine whether FAT/FRY ESCC is more sensitive to alpelisib.

Our study has some limitations. The data were derived from our bespoke panel, preventing us from comprehensively depicting the genetic features of the molecular subtypes described in our study. Nevertheless, such a widely used sequencing tool in clinical practice makes our results directly clinically relevant. Second, the predictive value of the 3-gene signature for predicting ICI efficacy could be partially explained by a higher TMB and neoantigen burden, which may lead to active antigen presentation by activated DCs and downstream antitumor immunity, but whether and how these Hippo pathway–related mutations reshape the tumor environment remain unknown.

These results represent our breakthroughs in understanding genetic alterations in ESCC from biological and clinical perspectives. We have identified and validated an ESCC molecular subtype with frequent Hippo-related mutations, a poor prognosis, and potential benefits to immunotherapy. With the popularization of large-panel sequencing in clinical practice, our findings will help clinicians make treatment decisions based on genomic features of patients.

## Methods

### Sample selection and sequencing.

The discovery cohort comprised 201 patients with available frozen tissues identified from the Biobank of Sun-Yat sen University Cancer Center (SYSUCC) according to our established criteria (see the note accompanying Supplemental Figure 1). Tumor purity was assessed by SYSUCC-authenticated pathologists, and only tissues with ≥50% tumor purity were included. Genomic DNA from frozen tissues was captured by our customized kit (SureSelect, Agilent) and sequenced on the Illumina NovaSeq 6000 platform. Our panel included 548 genes that were selected on the basis of previously identified mutation frequencies (2, 16–22).

To validate our findings from the discovery cohort, 70 frozen tissues from the biorepository of Guangdong Esophageal Cancer Institute were selected according to criteria used in the discovery cohort and were termed the validation cohort. Univariable Cox regression analysis was performed with bootstrap sampling in the discovery cohort, and 66 mutated genes associated with DFS or OS were included in the sequencing panel of the validation cohort. The library construction, sequencing, and bioinformatic analysis strategies were identical to those used in the discovery cohort.

### Bioinformatic analysis.

The clean reads were aligned to the human reference genome b37 using BWA. Variants were identified using Mutect2 (GATK-MuTect2 version v4.2.1) by comparing tumor samples with a normal sample pool. To overcome the difficulty of distinguishing somatic mutations and germline variants in the absence of matched normal samples, variant selection criteria were developed (Supplemental Methods).

### Unsupervised machine learning.

The log-rank test was applied to each mutated gene (mutant vs. WT), and all 59 prognosis-associated genes (*P*_DFS_ ≤ 0.1 or *P*_OS_ ≤ 0.1 and frequency ≥ 2%) were assembled into a binary gene-sample matrix for unsupervised clustering.

To identify ESCC subpopulations that shared similar prognosis-associated mutation patterns, NMF consensus clustering was used to cluster patients with similar mutation patterns. Another clustering algorithm, partitioning around medoids consensus clustering (29), was applied to repeat the generation of clusters. Venn plots and the kappa index were used to evaluate and visualize the consistency of clusters identified by the 2 algorithms. Other computational methods are detailed in Supplemental Methods.

### Analysis of external data sets.

GSE23400, GSE44021, and GSE161533 were processed by the R package *limma* to compare the expression differences of FRY between ESCC tumors and matched normal tissues. To validate the prognostic value of the 3-gene signature, genomic and survival data were extracted from associated studies (17, 20, 50). Clinical and genomic data from 2 pan-cancer cohorts and 1 NSCLC cohort were used to evaluate the capacity of the 3-gene signature in predicting the efficacy of immunotherapy (11–13).

To further characterize molecular features of the FAT/FRY subtype, we performed multiomics analyses based on the data from TCGA and GSE47404 (9, 51). The count data were processed by R package *edgeR* to identify differentially expressed genes (52). We used gene set enrichment analysis to identify enriched pathways (*P* < 0.05; FDR < 0.25). The relative composition of immune cells was evaluated by summarizing signals of marker genes (Supplemental Table 8) into the *z* score based on published methods (*GSVA* package) (53). SubMap analysis (54) was performed to measure the similarity of expression features between the FAT/FRY subtype tumors and responders of immunotherapies in 2 melanoma data sets and a urothelial cancer data set (42, 55, 56). The drug-response data (IC_50_) of 22 ESCC cell lines with Cancer Cell Line Encyclopedia mutational profiling data were retrieved from GDSC (57). A higher IC_50_ indicated a more resistant cell phenotype to the drug. Student’s 2-tailed *t* test was used to compare drug responses of the FAT/FRY mutant cell lines and WT cell lines.

### Data access.

Raw data will be uploaded to the Genome Sequence Archive in the BIG Data Center (https://ngdc.cncb.ac.cn/gsa), Beijing Institute of Genomics, Chinese Academy of Sciences, with accession code HRA000777. The public data sets used in this study can be retrieved from associated files of papers. The authenticity of the study was validated by the uploading of key raw data to the research data deposit public platform (http://www.researchdata.org.cn; approval RDD no. RDDB2021001611).

### Statistics.

All analyses were performed with R 4.0.2 and SPSS 25.0 (IBM Corporation). The *P* value for the survival curve was calculated from the log-rank test; all patients were followed up for death until December 31, 2019. The median follow-up time was 52.9 months (95% CI, 46.0–61.9, reverse Kaplan-Meier method), and 151 of 271 patients died during the follow-up period. Student’s 2-tailed *t* test or the Wilcoxon rank-sum test was used to assess associations between 2 groups of continuous variables, as appropriate. Fisher’s exact test was used to assess associations between categorical variables, including determining whether the oncogenic mutations in cancer-associated genes had a bias toward being “clonal.” Clonal events were deemed early events, and subclonal events were acquired later. The fixed (*I*^2^ < 50%) or random effects (*I*^2^ < 50%) model was used to pool the HRs of the molecular subgroup from 3 cohorts. All *P* values reported are 2 sided. The *P* value threshold for statistical significance was set at 0.05 unless otherwise specified.

### Study approval.

The Ethics Committee of Sun Yat-sen University Cancer Center approved the study protocol and waived the requirement for informed consent given the retrospective nature of the study (SZR2019-109).

## Author contributions

JF, JW, and HYconceived the study and were responsible for funding acquisition. ZM, XX, SF, and XW collected the data. JX provided critical instruction on the design of the analytical pipeline. ZM, HY, JX, and SF analyzed and interpreted the data. JY and ZM designed and performed the in vitro experiment. ZM, JY, JW, HY, and SF wrote and revised the manuscript, which was edited and approved by all the authors. The order of co–first authors was assigned according to discussions and votes by all the authors.

## Supplementary Material

Supplemental data

Supplemental tables 1-10

## Version 1 08/22/2022

Electronic publication

## References

[1] Hong Yang (2018). Neoadjuvant chemoradiotherapy followed by surgery versus surgery alone for locally advanced squamous cell carcinoma of the esophagus (NEOCRTEC5010): a phase III multicenter, randomized, open-label clinical trial. J Clin Oncol.

[2] Lin DC (2014). Genomic and molecular characterization of esophageal squamous cell carcinoma. Nat Genet.

[3] Cui Y (2020). Whole-genome sequencing of 508 patients identifies key molecular features associated with poor prognosis in esophageal squamous cell carcinoma. Cell Res.

[4] Turajlic S (2019). Resolving genetic heterogeneity in cancer. Nat Rev Genet.

[5] Gerstung M (2020). The evolutionary history of 2,658 cancers. Nature.

[6] Papaemmanuil E (2013). Clinical and biological implications of driver mutations in myelodysplastic syndromes. Blood.

[7] Cristescu R (2015). Molecular analysis of gastric cancer identifies subtypes associated with distinct clinical outcomes. Nat Med.

[8] Yu J (2015). Novel recurrently mutated genes and a prognostic mutation signature in colorectal cancer. Gut.

[9] Cancer Genome Atlas Research Network (2017). Integrated genomic characterization of oesophageal carcinoma. Nature.

[10] Kelly RJ (2021). Adjuvant nivolumab in resected esophageal or gastroesophageal junction cancer. N Engl J Med.

[11] Samstein RM (2019). Tumor mutational load predicts survival after immunotherapy across multiple cancer types. Nat Genet.

[12] Miao D (2018). Genomic correlates of response to immune checkpoint blockade in microsatellite-stable olid tumors. Nat Genet.

[13] Hellmann MD (2018). Genomic features of response to combination immunotherapy in patients with advanced non-small-cell lung cancer. Cancer Cell.

[14] Charoentong P (2017). Pan-cancer immunogenomic analyses reveal genotype-immunophenotype relationships and predictors of response to checkpoint blockade. Cell Rep.

[15] Mai Z (2021). Integration of tumor heterogeneity for recurrence prediction in patients with esophageal squamous cell cancer. Cancers (Basel).

[16] Dai W (2017). Whole-exome sequencing reveals critical genes underlying metastasis in oesophageal squamous cell carcinoma. J Pathol.

[17] Sawada G (2016). Genomic landscape of esophageal squamous cell carcinoma in a Japanese population. Gastroenterology.

[18] Qin HD (2016). Genomic characterization of esophageal squamous cell carcinoma reveals critical genes underlying tumorigenesis and poor prognosis. Am J Hum Genet.

[19] Zhang L (2015). Genomic analyses reveal mutational signatures and frequently altered genes in esophageal squamous cell carcinoma. Am J Hum Genet.

[20] Song Y (2014). Identification of genomic alterations in oesophageal squamous cell cancer. Nature.

[21] Gao YB (2014). Genetic landscape of esophageal squamous cell carcinoma. Nat Genet.

[22] Agrawal N (2012). Comparative genomic analysis of esophageal adenocarcinoma and squamous cell carcinoma. Cancer Discov.

[23] Irie K (2020). Furry protein suppresses nuclear localization of yes-associated protein (YAP) by activating NDR kinase and binding to YAP. J Biol Chem.

[24] Liu Y (2019). Fry is required for mammary gland development during pregnant periods and affects the morphology and growth of breast cancer cells. Front Oncol.

[25] Sanchez-Vega F (2018). Oncogenic signaling pathways in the cancer genome atlas. Cell.

[26] Andre F (2019). Alpelisib for PIK3CA-mutated, hormone receptor-positive advanced breast cancer. N Engl J Med.

[27] Yan T (2019). Multi-region sequencing unveils novel actionable targets and spatial heterogeneity in esophageal squamous cell carcinoma. Nat Commun.

[28] Gaujoux R, Seoighe C (2010). A flexible R package for nonnegative matrix factorization. BMC Bioinformatics.

[29] Wilkerson MD, Hayes DN (2010). ConsensusClusterPlus: a class discovery tool with confidence assessments and item tracking. Bioinformatics.

[30] Liu G (2020). AHNAK2 promotes migration, invasion, and epithelial-mesenchymal transition in lung adenocarcinoma cells via the TGF-β/Smad3 pathway. Onco Targets Ther.

[31] Zhang H (2020). Exome sequencing identifies new somatic alterations and mutation patterns of tongue squamous cell carcinoma in a Chinese population. J Pathol.

[32] Davis TA (2014). AHNAK: the giant jack of all trades. Cell Signal.

[33] Li Z (2018). Loss of the FAT1 tumor suppressor promotes resistance to CDK4/6 inhibitors via the Hippo pathway. Cancer Cell.

[34] Martin D (2018). Assembly and activation of the Hippo signalome by FAT1 tumor suppressor. Nat Commun.

[35] Sondka Z (2018). The COSMIC Cancer Gene Census: describing genetic dysfunction across all human cancers. Nat Rev Cancer.

[36] Katoh M (2012). Function and cancer genomics of FAT family genes (review). Int J Oncol.

[37] Zhang Y (2018). Hippo signaling in the immune system. Trends Biochem Sci.

[38] Lu S (2019). Comparison of biomarker modalities for predicting response to PD-1/PD-L1 checkpoint blockade: a systematic review and meta-analysis. JAMA Oncol.

[39] Ballman KV (2015). Biomarker: predictive or prognostic?. J Clin Oncol.

[40] Ayers M (2017). IFN-γ-related mRNA profile predicts clinical response to PD-1 blockade. J Clin Invest.

[41] Jerby-Arnon L (2018). A cancer cell program promotes T cell exclusion and resistance to checkpoint blockade. Cell.

[42] Hugo W (2016). Genomic and transcriptomic features of response to anti-PD-1 therapy in metastatic melanoma. Cell.

[43] Lu T (2020). Tumor neoantigenicity assessment with CSiN score incorporates clonality and immunogenicity to predict immunotherapy outcomes. Sci Immunol.

[44] Zheng X (2018). Prognostic role of tumor-infiltrating lymphocytes in esophagus cancer: a meta-analysis. Cell Physiol Biochem.

[45] Xie J (2021). A novel platelet-related gene signature for predicting the prognosis of triple-negative breast cancer. Front Cell Dev Biol.

[46] Pastore A (2015). Integration of gene mutations in risk prognostication for patients receiving first-line immunochemotherapy for follicular lymphoma: a retrospective analysis of a prospective clinical trial and validation in a population-based registry. Lancet Oncol.

[47] Markham A (2019). Alpelisib: first global approval. Drugs.

[48] Tumaneng K (2012). YAP mediates crosstalk between the Hippo and PI(3)K–TOR pathways by suppressing PTEN via miR-29. Nat Cell Biol.

[49] Xu W (2018). PTEN lipid phosphatase inactivation links the hippo and PI3K/Akt pathways to induce gastric tumorigenesis. J Exp Clin Cancer Res.

[50] Liu J (2018). An integrated TCGA pan-cancer clinical data resource to drive high-quality survival outcome analytics. Cell.

[51] Sawada G (2015). An integrative analysis to identify driver genes in esophageal squamous cell carcinoma. PLoS One.

[52] Robinson MD (2010). edgeR: a Bioconductor package for differential expression analysis of digital gene expression data. Bioinformatics.

[53] Lee E (2008). Inferring pathway activity toward precise disease classification. PLoS Comput Biol.

[54] Hoshida Y (2007). Subclass mapping: identifying common subtypes in independent disease data sets. PLoS One.

[55] Chen PL (2016). Analysis of immune signatures in longitudinal tumor samples yields insight into biomarkers of response and mechanisms of resistance to immune checkpoint blockade. Cancer Discov.

[56] Snyder A (2017). Contribution of systemic and somatic factors to clinical response and resistance to PD-L1 blockade in urothelial cancer: an exploratory multi-omic analysis. PLoS Med.

[57] Yang W (2013). Genomics of drug sensitivity in cancer (GDSC): a resource for therapeutic biomarker discovery in cancer cells. Nucleic Acids Res.

