# Supplementary material for: Inactivation of Hippo pathway characterizes a poor-prognosis subtype of esophageal cancer
Source: JCI Insight. 2022 Aug 22;7(16):e155218. doi: 10.1172/jci.insight.155218 (PMC9462502; doi:10.1172/jci.insight.155218)
Supplement: Supplemental data [file jciinsight-7-155218-s076.pdf]

Catalogue

Supplementary methods......2
Sample selection......2
Follow up of patients and collection of clinical data......2
Gene panel design and sequencing......2
Data generation of validation cohort......3
Tumor purity determinant......3
Reads alignment and Variant calling......3
Detection of significantly mutated genes......4
Sanger sequencing......4
Analysis of copy number alterations......5
Oncogenic mutation annotataion......5
Inference of temporal order of oncogenic mutations......5
Estimation of cancer cell fraction......6
Unsupervised machine learning......6
Analysis of external datasets......6
Analysis of drug response for different ESCC subgroups......7
Statistical analysis......7
RNA extraction, reverse transcription and quantitative PCR......8
Cell Lines and Culturing......8
Small Interfering RNA Mediated Gene Knockdown......8
Overexpression of Truncated protein of FRY......8
Western Blotting......9
Cell Proliferation Assays......9
Immunohistochemistry staining and digital analysis......9
Supplementary figure......10
Figure S1 Diagram of samples selection workflow.......11
Figure S2 Overview of our analysis schedule......11
Figure S3 Lolliplot of frequently mutated genes......12
Figure S4 Correlations of expression of several Hippo-associated genes and FRY in three microarray datasets.13
Figure S5 Association of the three-gene signature with patient prognosis......16
Figure S6 Survival curves of patients according to three-gene signature stratified by clinicopathological parameters......16
Figure S7 Comparisons of the sensitivity and specificity for prediction of disease progression and deathby the three-gene signature and the TNM stage.......17
Figure S8 Prognostic value of the three-gene mutation signature detected by different methods in TCGA cohort. 17
Figure S9 Representative images of CD8 immunohistochemistry of ESCC tissues from FAT/FRY muatant and wild type patients.......18
Figure S10 FAT/FRY signature is prognosticator instead of predictor of ajuvant therapy.......18
Figure S11 Prognostic value of genomic and transcriptomic features of FAT/FRY subgroup tumors......19
Table S1 Detailed comparison of clinical variables between discovery cohort and independent validation cohort 20
Table S5 Multivariable Cox regression analysis of the three-gene signature and clinicopathological factors......21
Table S6 Detailed comparison of clinical variables between "FAT/FRY" group and wild type group across the 271 ESCC patients......22
Table S8 Publicly-available gene signatures used in the study......23

Table S11 Primers used in our qRT-PCR experiment.. 23

Supplementary methods

Sample selection

All the samples in our study came from surgical specimens were stored at -80 ℃ in the biobank of Sun-Yat Sen University Cancer Center. All patients underwent esophagectomy, achieved complete resection without receiving neoadjuvant therapy and experienced lymph node metastasis. 201 fresh frozen samples were chosen following our established criteria (Figure S1).

Inclusion criteria contained:

(1) Patients whose age⩾18 years, preoperative KPS score⩾90;

(2) Pathological diagnosis as esophageal squamous cell carcinoma and tumor located in thoracic segment of esophagus;

(3) R_0 resection via thoracic approach and standard lymph node excision;

(4) Neo-adjuvant treatment naïve;

(5) Patients with lymph node metastasis confirmed by pathological diagnosis

Exclusion criteria contained:

(1) Patients with secondary primary tumor;

(2) Patients died within 30 days after surgery or died of post-operation complication;

(3) Patients missing essential clinical information, such as age, sex, operation record, pathological diagnosis and follow-up data.

(4) Patients with distant metastasis by PET-CT.

Follow up of patients and collection of clinical data

Patients were followed up through regular outpatient service four times per year within the first year after surgery, twice per year from the second to the fifth year, and once a year after five years. Regular examination included physical examination, blood and biochemical routine examination, tumor biomarker (SCC and CEA), endoscopy and CT. Demographic and clinical data were extracted from our clinical database.

Clinical endpoint data was prepared following the common used criteria[1]. Disease free survival (DFS) is defined as the shortest period from the date of surgery to the date of first tumor recurrence event with radiological or pathological confirmation. The censored time is from the date of surgery to the last contact date or date of death. Overall survival (OS) is the longest period from the date of surgery until the date of death at any cause. The censored time is from the date of surgery to the date of last contact. Comprehensive pathological staging was conducted by experienced pathologists following the 8th edition of AJCC cancer staging manual.

Gene panel design and sequencing

Mutation data were downloaded from supplementary materials of published result[2-9]. We calculated mutation frequency for each gene based on WGS/WES data from 589 patients. In order to gain in-depth insight of mutations of ESCC, we brought genes with mutation frequency above 2%

into our panel list. Ultimately, all exons of 548 selected genes covering 5.731 Mbp were used to design complementary probes for library construction.

AllPrep DNA Universal Kit (SureSelect, Agilent, Santa Clara, USA) was used to extract DNA from frozen fresh tissues (purity>50%, median: 70%). DNA was quantified and quality controlled by Qubit 2.0 and Agarose gel electrophoresis assay prior to library construction. DNA was broken into 180-280 bp and all exons of 548 genes were captured using Agilent SureSelect XT Custom Kit. After PCR amplification and quality control by Agilent 2000, DNA library was sequenced using paired-end 150 bp on Illumina Hiseq platform. The average sequencing depth of coverage on target regions was 1070X (range: 690X—1616X), and 95% of targets were covered by 100 reads.

Data generation of validation cohort

To verify the prognostic value of our findings, an independent cohort was recruited from the biorespository of Guangdong Esophageal Cancer Institute (GECI). The tumor samples of GECI were collocted from thoracic division of Sat-Yat Sen University Cancer Center. Following the same inclusion and exclusion criteria used in discovery cohort, 70 samples with qualified frozen tissues were selected from 335 cases in our biorespository. Detailed comparison of clinical information between 201 patients in discovery cohort and 70 patients in validation cohort was listed in Table S1. Univariable Cox regression was performed with 2000 boostrap sampling in discovery cohort, and 66 genes associated with DFS or OS were included in the sequencing panel of validation cohort. The design of captured probe and library construction of samples were identical to those applied in discovery cohort. DNA library was sequenced using paired-end 150 bp on Illumina Hiseq 2500 platform. The median coverage of depth in validation cohort was 1026X (range: 515X-1648X). The clean data of validation cohort was then processed following the same bioinformatic analysis pipeline.

Tumor purity determinant

All samples underwent pathological review via frozen section. A tissue section was created with two H&E slides (termed as top and bottom): a 4 μm frozen section (top slide) was cut, 20 mg of tumor tissue was shaved from the tissue for library construction, then a second 4 μm frozen section was cut (bottom slide). An H&E stain was conducted on both slide tissue sections. SYSUCC-authenticated pathologist conducted diagnosis verification and turmor purity assessment. Pathologist initially screened the slide in low magnification to determine the microscopic morphology, then magnified to 20X and reviewed 10 representive fields on each slide. The tumor purity was derived from the proportion of tumor nuclei compared to total nuclei present on the slide. The tumor purity of each sample was the aveage level of purities in both top and bottom slides. For quality control, random review of 20% of slides was conducted by a second pathologist to confirm the results. If the results of second review were off by 10%, the sample would be assessed again.

Reads alignment and Variant calling

Clean reads were obtained after filtering out low-quality reads and adapters from raw reads. The clean reads were aligned to human reference genome b37 using BWA[10] and deduplicated using SAMBLASTER[11]. Variants (SNVs and small Indels) were identified using Mutect2 by comparing tumor samples with an unmatched normal sample pool[12]. All putative variants were annotated

using ANNOVAR[13]. To account for the absence of matched control, a custom variant sifting pipeline was developed:

(1) Removal of variants located within low-coverage (<10X) regions and variants with less than 3 mutant reads

(2) Removal of variants whose allele fraction is 1

(3) For variants with well-characterized annotation in COSMIC[14], removal of known polymorphisms reported among 1000 Genome, Exome Aggregation Consortium data[15] or in-house database at frequency >0.1

(4) For vatiants without annotation in COSMIC, removal of variants recorded in dbSNP, variants with frequency above 0.003 in 1000 Genome data, variants with frequency above 0.01 in in-house database and variants with frequency above 0.001 in Exome Aggregation Consortium data

(5) Removal of germline variants present in any of normal control.

After filtering the probably germline variants, the remaining mutations were used for further analysis in our study.

Detection of significantly mutated genes

According to Darwinian evolution, mutations that endowed tumor cells survival advantage will accumulate during tumor development. Following this principle, we statistically evaluated three types of features extensively observed in driver genes: 1) mutation recurrence; 2) deleterious mutation enrichment; 3) Mutation hotspots. The methodological details were as follow[3].

(1) Mutation recurrence test.

To test whether mutations in a gene frequently occurred across sample, OncodriveCLUST was employed to evaluate mutation recurrence while considering gene length, distribution of mutations across gene loci and background mutation rate[16].

(2) Deleterious mutation enrichment test.

A genuine driver gene is prone to undergo mutational hits that damage the protein function than non-driver gene does. In this study, nonsense, frameshift, splice site mutations and missense mutations scored under 0.05 by SIFT[17] were considered deleterious to protein function. For each gene, we assumed the numbers of deleterious mutations and all mutations as the number of successes and trials, respectively. Then we constructed a binomial model to evaluate the enrichment of deleterious mutation. As for the probability in binomial model, we used the ratio of deleterious mutations to all mutations in non-recurrently mutated genes.

(3) Mutation cluster

Mutational hotspot is a strong indicator of positive selection[18].Rather than nucleotide-level analysis, we applied an algorithm to detect amino acid residue-level hotspots[19]. Hotspot in the algorithm was defined as an amino acid position in protein-coding gene mutated more frequently, corresponding to mutations located in the same codon.

Several significant mutated genes were identified at the threshold of $adj.P_{OncodriveCLUST} \leqslant 0.1$ and $P_{binomial} \leqslant 0.05$: TP53, FRY, ZNF750, NFE2L2, GRIN2B and FCGBP.

Sanger sequencing

110 mutations were randomly selected for validation. Because common detection threshold of mutations by Sanger sequencing is 10% of VAF[20, 21], we filter 59 mutations with a frequency of over 10%. Among these mutations, 3 cases were failed due to difficulty of PCR amplification. Finally,

Sanger sequencing succeed in 56 cases. 98.2% (55/56) of mutations detected by NGS were verified by Sanger sequencing. Mutations of FRY gene in cell lines were also detected by Sanger sequencing. Sequences of primers would be available upon request.

Analysis of copy number alterations

Copy number analysis was conducted using CNVkit[22] which was designed specific for targeted sequencing data. In brief, the read counts of 50 normal samples were normalized and integrated into a pool reference. Then targeted reads and nonspecifically captured off-target reads from tumor samples were used to infer somatic copy number alterations. The algorithm also adjusted the bias that leads to sequencing read depth: GC content, target size, repetitive sequences. Copy number alterations (CNAs) were inferred following default parameters and adjusted by tumor purity.

Oncogenic mutation annotataion

Each somatic mutation was annotated as oncogenic or unknown. Detailed annotation parameters are listed as follows, which is similar to the criteria of oncogenic mutation in published works[23, 24]:

(1) Oncogenic

a. Known oncogenic mutations reported in the literature[2-9, 14, 25-29];
b. Hotspot mutations ($n \geqslant 3$) that located in known cancer associated genes;
c. Truncating variants (nonsense mutations, splice site mutations, nonstop mutations and frameshift insertions or deletions) in cancer associated genes;

(2) Unknown

a. Mutations outside of frequent oncogenic mutations in genes with known oncogenic mutations;
b. Mutations in genes whose role in ESCC is not established yet.

Inference of temporal order of oncogenic mutations

The mutation order analysis was conducted using the previous described methods[23, 24]. Before testing whether one mutation occurred earlier the others, the variant allelic frequency (VAF) of each oncogenic mutation was calibrated by copy number of loci at which mutations located according to the method described previously[30]. In brief, VAF of homozygous mutations and mutations of genes located on chromosome X in male cases were reduced to the half of the raw data. VAF of hemizygous mutations were recalculated based on the formula as "Adjusted VAF=x/(1+x)", while x refers to raw VAF. Adjustment was not required for heterozygous mutations.

To test whether there existed evidence that two oncogenic mutations within a patient were present in the same fraction of cells, we apply fisher exact test using the adjusted VAF. We set a significance threshold of $P \leqslant 0.05$ at determining whether clonal heterogeneity existed in a given patient. Then we employed the "pigeonhole" principle to reconstruct temporal precedences of oncogenic mutations, only including those pair comarisons with clear phylogenetic relationship[31]. From the set of genes in which at least 5 precedences were observed, we utilized Bradley-Terry model[32] (package: BraleyTerry2) using penalized maximum likelihood to the observed precedences[23, 24]. Quasi standard error was computed using 'qvcalc' package so that comparison between any pair of genes was readily made, not just the comparison with the reference gene.

Estimation of cancer cell fraction

Following the algorithm described previously[33, 34], we computed the posterior probability distribution over cancer cell fraction (CCF) of mutations to estimate their clone status. Let b denoted the number of reads supporting such mutation, d denoted the total reads covering the mutation locus, ρ referred to the tumor purity, c_t and c_n referred to the copy number of the gene locus at that base in the tumor and normal genome respectively. The expected allele-fraction f(c) of a mutation present in one copy in a fraction c of cancer cells was calculated by f(c) = $c * \frac{\rho}{(1-\rho)c_n+\rho c_t}$, with $c \in [0.01,1]$. Then P(c) $\propto$ Binomial(bld, $f(c)$) assuming a uniform prior on c. The distribution over CCF was obtained by calculating values over a regular grid of 100 c values and normalizing. Mutations were classified as clonal on the ground of the probability that the CCF exceed 0.85. A probability threshold of 0.5 was used in our study.

To infer the proportion of tumor cells carrying a given mutation, we used the following formula[31, 35]:

$$\mathrm{CCF} = \min\left(1, \frac{b}{d} * \frac{(1-\rho)c_n+\rho c_t}{\rho}\right)$$

Unsupervised machine learning

Prognosis related genes were selected as below for further clustering. To reduce false negative rate and enhance statistics power, two follow-up end points (DFS and OS) and a relatively loose significant threshold were used to generate gene-sample matrix. Log-rank test was applied to each mutated gene (mutant VS wild type), and all 59 prognosis-associated genes ($P_{DFS} \leq 0.1$ or $P_{OS} \leq 0.1$ and frequency≥2%) were assembled into a binary gene-sample matrix. None of mutation in 59 genes was observed in four patients, genomic data of these four patients was excluded from gene-sample matrix due to the mathematical constraints of nonnegative matrix factorization (NMF).

To dig out ESCC subgroups that shared similar mutational patterns associated with prognosis, NMF was used to cluster patients with similar mutation patterns (package: NMF). The number of cluster k=3 was chose as it yielded a high cophenetic coefficient and effectively decomposed the matrix[36]. To examine the robustness of the above NMF-based clusters, another entirely different clustering algoritmn, partitioning around mediods consensus clustering[37], was applied (package: ConsensusPlus). The number of clusters k=3 was picked by inspecting the bimodality of CDF curves and progression of area under CDF curves[38]. Venn plot and Kappa index （package: irr）were used to evaluate and visualize the consistency of clusters identified by two algorithms.

Analysis of external datasets

To evaluate whether FRY was dysregulated in ESCC, paired t test was used to compare the mRNA levels of FRY in tumors and matched normal and paratumor tissues in three available ESCC datasets, GSE23400, GSE44021 and GSE161533. Three datasets were further merged and batch effects were adjusted using Limma package. To further discover the correlation of FRY and Hippo pathway, we calculated the spearman correlation coefficients of FRY and Hippo target genes. Taking advantage of the reverse-phase protein arrays (RPPA) data from the TCGA project, we measured the relationship of FRY and YAP1, the only protein of Hippo pathway in the RPPA.

To validate the prognostic value of our three-gene signature, two independent datasets were used to perform survival analysis[6, 39]. As for TCGA cohort, WES data were downloaded from

UCSC Xena. Mutations were called by “Muse”, ”Mutect”, “SomaticSniper” and “Varscan”. Mutations only detect by more than two callers were used for further analysis. Patients were grouped according to their mutation status of FAT1, FAT3 and FRY. Notably, the lack of accurate N stage information in the TCGA cohort prevented us to further subdivide patients by status of their lymph node metastasis. Note that the prognostic value of the three-gene mutation signature was insensitive to choice of mutation callers (Figure S6). Similar analysis was performed in Song’s cohort[6], and patients were subdivided by their positive lymph node status compatible with our discovery cohort.

To further characterize molecular features of the “FAT/FRY” subtype, we performed multiomics analysis based on the data from TCGA and GSE47404. For transcriptome analysis, the read count data were transformed to TMM by the R package edgeR to identify differentially expressed genes[40]. For microarray data, differentially expressed genes was detected by package “limma”. We used gene set enrichment analysis to identify enriched pathways in the molecular subtypes (package: Clusterprofiler). To dissect the composition of tumor environment, we calculated the z-score of immune cells to represent their relative composition in ESCC micro-environment using package “GSVA” [41-43]. Briefly, this method could evaluated activities of pathway by automatically catched a subset of genes in the pathway whose combined expression delivers optimal discriminative power for the disease phenotype. Marker genes representing each immune cell subset could be sourced from published papers (Table S8).

To assess the capacity of our molecular subtypes to predict immunotherapy responses, we applied subclass mapping (SubMap) to measure the similarity of transcriptome profile of our molecular subtypes and that of the groups with different responses in the immunotherapy cohort. The SubMap algorithm evaluates the extent of commonality of the different subgroups in independent datasets. Permutation-based P-values were used to evaluate the similarity, and the lower the P-values were, the higher the similarity. We applied SubMap (GenePattern) to measure the similarity of ESCC molecular subtypes with different responses of patients from two melanoma cohorts and a urothelial cancer cohort treated with immune checkpoint inhibitors[44-46].

Furthermore, we evaluated the predictive capacity of FAT/FRY signature in immunotherapy cohorts. Due to the lack mature sequencing data of ESCC, we chose to perform survival analysis in a pancancer cohort with microsatellite stable (MSS) tumors and a NSCLC cohort as an alternative[47-49], because most of ESCC were MSS[50] and the genetic background of ESCC were similar to that of NSCLC[51].

Analysis of drug response for different ESCC subgroups

The drug response data (half maximal inhibitory concentration, IC50) of 22 ESCC cell lines with Cancer Cell Line Encyclopedia mutational profiling data were retrived from Genomics of Drug Sensitivity in Cancer[52]. The higher IC50 indicated a more resistant phenotype of the cell. Student t test was used to compared drug responses of the FAT/FRY mutant cell lines and those with wild type.

Statistical analysis

All analyses were performed in R 4.0.2 and SPSS 25.0 (IBM Corporation). The P value for the survival curve was calculated from the log-rank test. Student’s t test or the Wilcoxon rank-sum test was used to test for an association between two groups of continuous variables as appropriate. Paired t test was used to compare the mRNA levels of FRY in tumors and matched normal tissues in three available ESCC datasets, GSE23400, GSE44021 and GSE161533. Fisher’s exact test was

used to test for an association between categorical variables, including determining whether the oncogenic mutations of cancer-associated genes had a bias towards being “clonal”. Clonal events were deemed early events and subclonal events were acquired relatively later. The fixed (I^2<50%) or random effects ($I^2 \geq 50\%$) model was used to pool the HR of the molecular subgroup from three cohorts using “meta” package. We performed area under the curve (AUC) for the receiver operating characteristic (ROC) analysis to compare sensitivity and specificity for prediction of death and relapse at different cutoff times (2, 3 and 4 years) by our molecular subgroups and the AJCC 8th TNM stage using “timeROC” package. Similar analysis was performed to compare the performance of the three-gene signature and TMB in predicting post-immunotherapy outcome. All P values reported are two-sided. The P value threshold for statistical significance was set at 0.05. Several packages, including “ggplot2”, “ggsci”, “ggtheme”, “survival”, “maftools”, “trackviewer”, “GridExtra” and “VennDiagram” was used for data visuallization. To visualize the mutation profile of genes, the protein sequence annotations were downloaded from Uniprot database (https://www.ebi.ac.uk/proteins/api/).

RNA extraction, reverse transcription and quantitative PCR

Among the 201 patients in the discovery set, 90 fresh frozen tumors were available. Reasons for the absence of samples included the exhaustion of samples and prohibition of obtaining samples from the biobank due to their age. Total RNA was extracted from clinical samples using TRIzol reagent (Invitrogen) according to the manufacturer’s instruction. cDNA was synthesized from 1 μg of total RNA using RevertAid First Strand cDNA Synthesis Kit (Thermo Scientific). cDNA was subjected to quantitative real-time PCR (qRT-PCT). GAPDH was used as an internal control. The primers used in this study are shown in Table S11. QRT-PCR was performed using the Power SYBR Green PCR Master Mix (Applied Bio systems) and LightCycler480 PCR system (Roche Diagnostics).

Cell Lines and Culturing

Human ESCC cell lines KYSE30, KYSE410 were all preserved in State Key Laboratory Of Oncology In South China. All of these cells were cultured in DMEM medium supplemented with 10% fetal bovine serum and 1% antibiotics (100 mg/mL streptomycin and 100 units/mL penicillin) at 37 ℃ in a humidified incubator under 5% CO2 condition.

Small Interfering RNA Mediated Gene Knockdown

Depletion of gene expression was performed by transfecting cells with small interfering RNA (siRNA) oligonucleotides directed against the following target sequences: FRY-si1 ：GCAGGACCCTTCAGCATTA; FRY-si2：GCTACAACTACTTCGAATT. Transfection steps were performed following the manufacture’s protocols, using Lipofectamine RNAiMAX (Invitrogen, USA).

Overexpression of Truncated protein of FRY

The plasmid containing human partial length FRY cDNA (1-957) was amplified by PCR and cloned into pcdna3.1 vector and linked with a HA tag at the N-terminal. Transfection steps were performed following the manufacture’s protocols, using Lipofectamine 3000 (Invitrogen, USA)

Western Blotting

Cell lysate was prepared using a RIPA Lysis Buffer (Millpore, USA), and the protein concentration was measured using a BCA Protein Assay Kit (keyGEN BioTECH, China). Cell lysates were separated by 6%–10% sodium dodecyl sulfate (SDS)– polyacrylamide gel electrophoresis (PAGE) gel electrophoresis and transferred to a PVDF membrane. After blocking with 5% skim milk, the membrane was incubated with one of the following primary antibodies under 4℃ overnight: FRY (LS-C343004, mouse; LSBio, Germany), HA (3724, rabbit; CST, USA), Vinculin (13901, rabbit; CST, USA), Tubulin (3873, mouse; CST, USA), and then incubated with the species-specific secondary antibodies for one hour under room temperature. Finally, the membrane was incubated with Western Blotting Substrate（Thermo Scientific，USA）and detected by ChemiDoc Touch imaging system (Biorad，USA).

Cell Proliferation Assays

For cell viability assay, cells were seeded into a 96-well plate at 103 cells per well and cultured at 37 ℃. For each day, 10% (volume/volume) CCK-8 (Dojindo, Kumamoto, Japan) was added to the culture medium and incubation lasted for 1 hour. Cell viability was monitored by measuring absorbance at 450 nm using a Microplate Reader (MD SpectraMax PlusPower 384, USA). The experiment was performed in quintuplicate and repeated twice.

Immunohistochemistry staining and digital analysis

Previous immunogenomic analyses from both TCGA-ESCC and GSE47404 datasets show that CD8+ tumor inflitrated lymphocytes (TILs) were more abundant in FAT/FRY mutant ESCC. Therefore, immunohistochemistry(IHC) analysis was performed for CD8 (ZA-0508-3.0, ZSGB-BIO) to evaluate the CD8+ TIL inflitrations of 170 patiants with available tumor tissue slides from the discovery cohort. Reasons for absence of tumor slides included exhaustion of FFPE slides and absence of tumors in the slides. Polaris digital slides scanner (Akoya Biosciences, USA) was used to scan the slides and HALO digital pathological platform was used to quantify the density of CD8+ cells. The tumor regions wes mannually annotated by an experienced pathologist and the The RGB signal of the immunostaining markers was recognized by the multiplex IHC mode of HALO software and the cutoff of the signal was calibrated by the author (MZH). The positive cell densities of the tumor area was calculated as the number of positive cells divided by the tumor area measurements (cell counts/mm2). The proportions of CD8+ cells were also calculated to parallelly evaluate the abundance of CD8+ TILs, which was not affected by the size of annotated areas. Wilcoxon rank sum test was performed to compare the density of CD8+ cells between FAT/FRY mutant and wild type tumors.

Supplementary figure

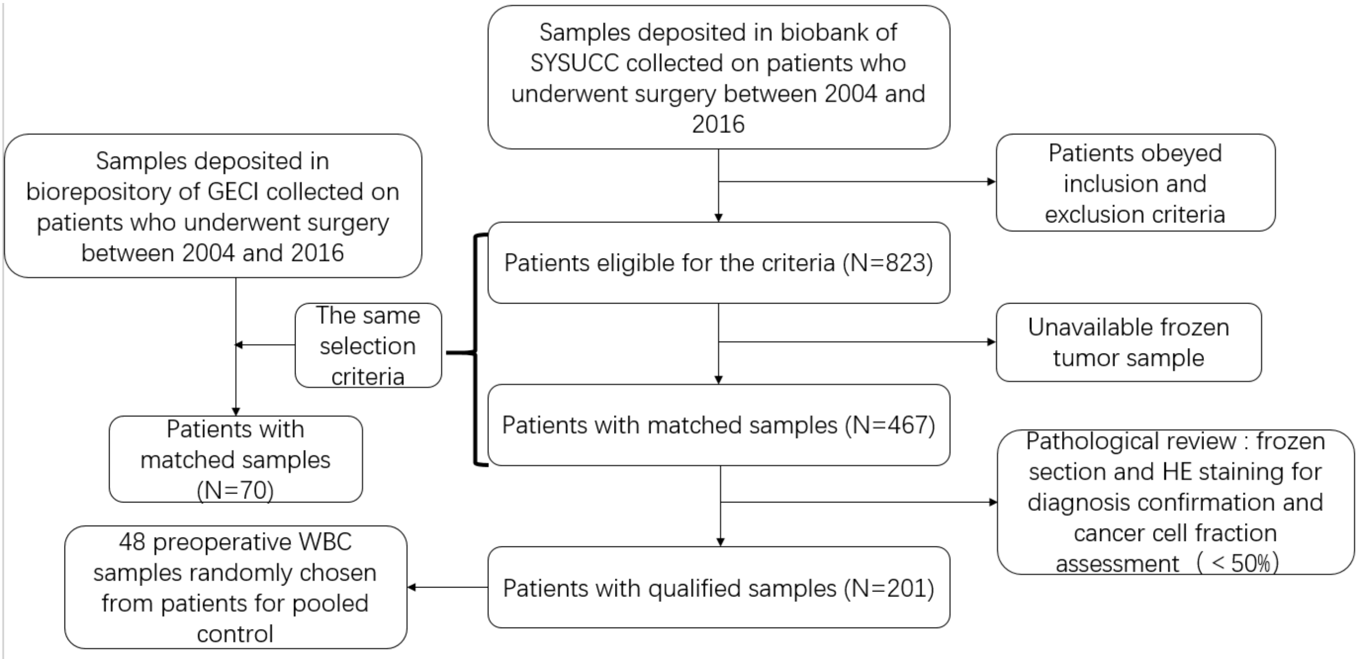

Figure S1 Diagram of samples selection workflow. Patients in discovery and validation cohorts were selected by the same criteria.

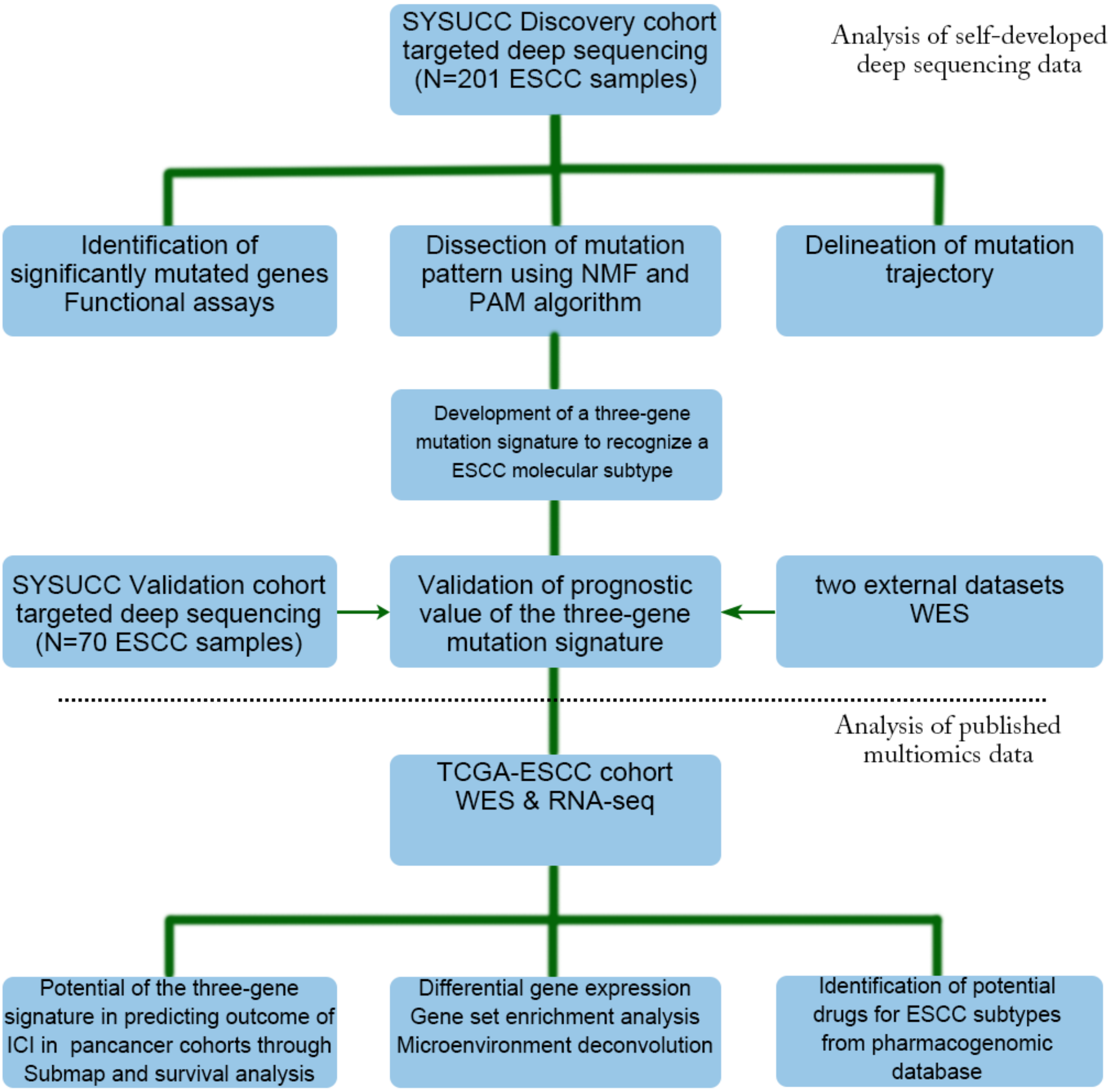

Figure S2 Overview of our analysis schedule

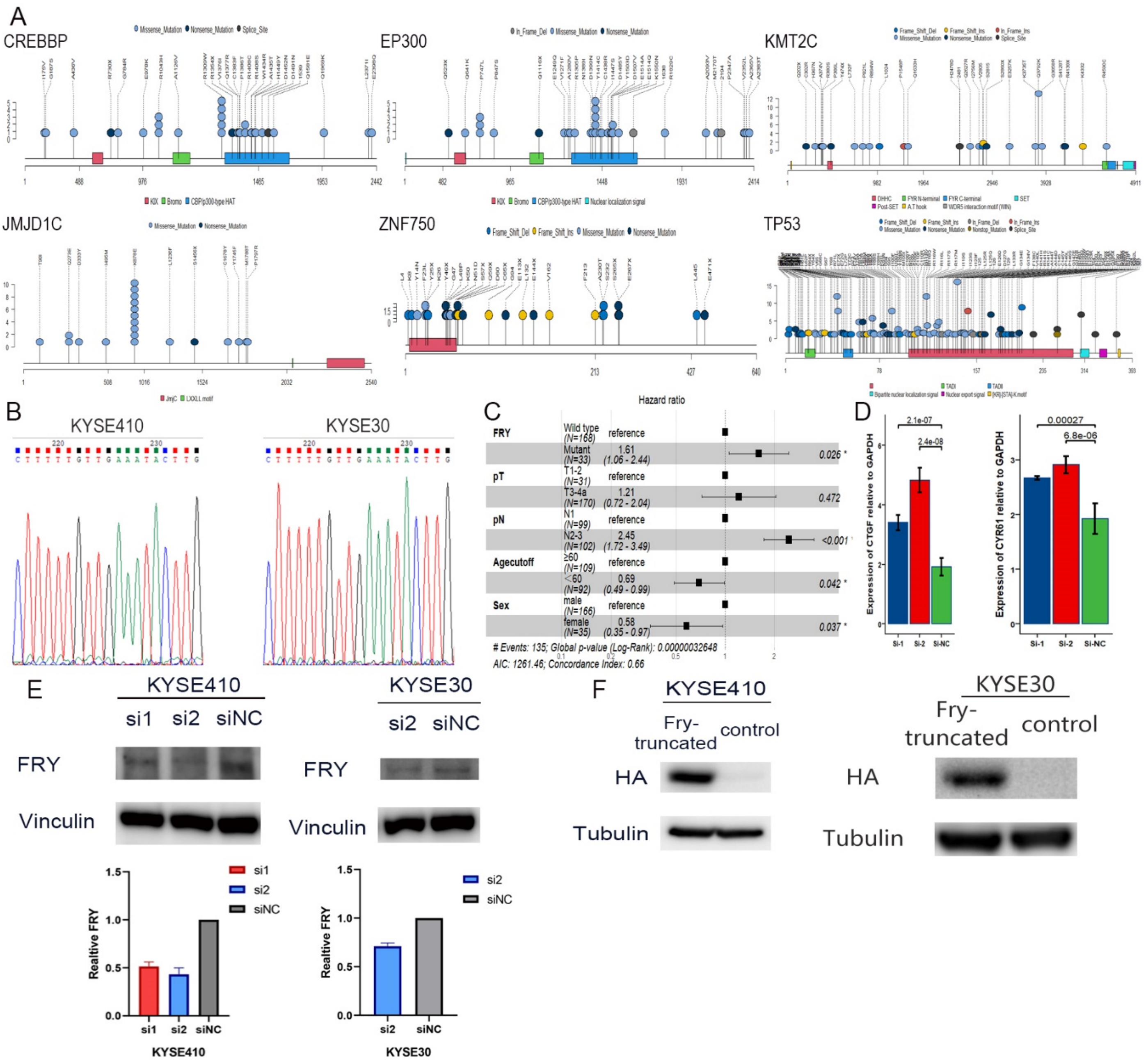

Figure S3 Lolliplot of frequently mutated genes. (A) Mutational hotspots accumulated in ESCC associated genes, including histone modifiers EP300, CREBBP, KMT2C and JMJD1C. (B) Sanger sequencing identified somatic mutations of FRY in ESCC cell lines. No FRY mutations were detected in KYSE410 and KYSE30 cell lines. (C) Multivariable Cox regression analysis of mutation status of FRY and clinicopathological variables. (D) The mRNA levels of CTGF and CYR61 in two RNAi KYSE410 cells and the control KYSE410 cells. (E) Western blot and quantitative analysis of knock down efficiency of FRY in KYSE410 and KYSE30 cells. (F) Western blot analysis of overexpression of hotspot mutation FRY p.E319X. Data represent mean ± SEM from 3 independent experiments and each had three replications

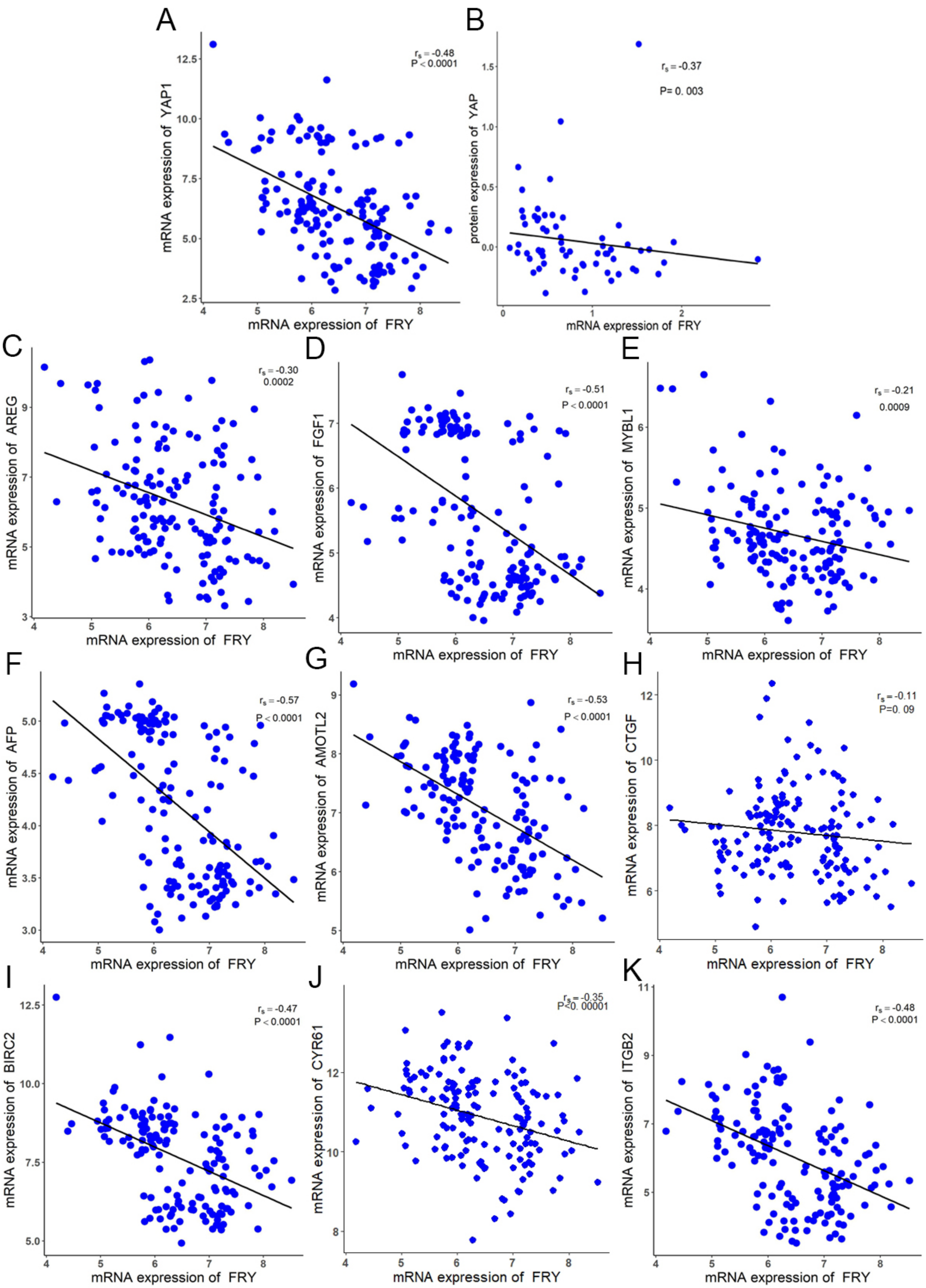

Figure S4 Correlations of expression of several Hippo-associated genes and

FRY in three microarray datasets. Negative correlation of FRY and YAP1 and its target genes in the combined microarray datasets (A, C-L). (B) Correlation of mRNA levels of FRY and protein expression of YAP1 in the TCGA-ESCC RPPA datasets.

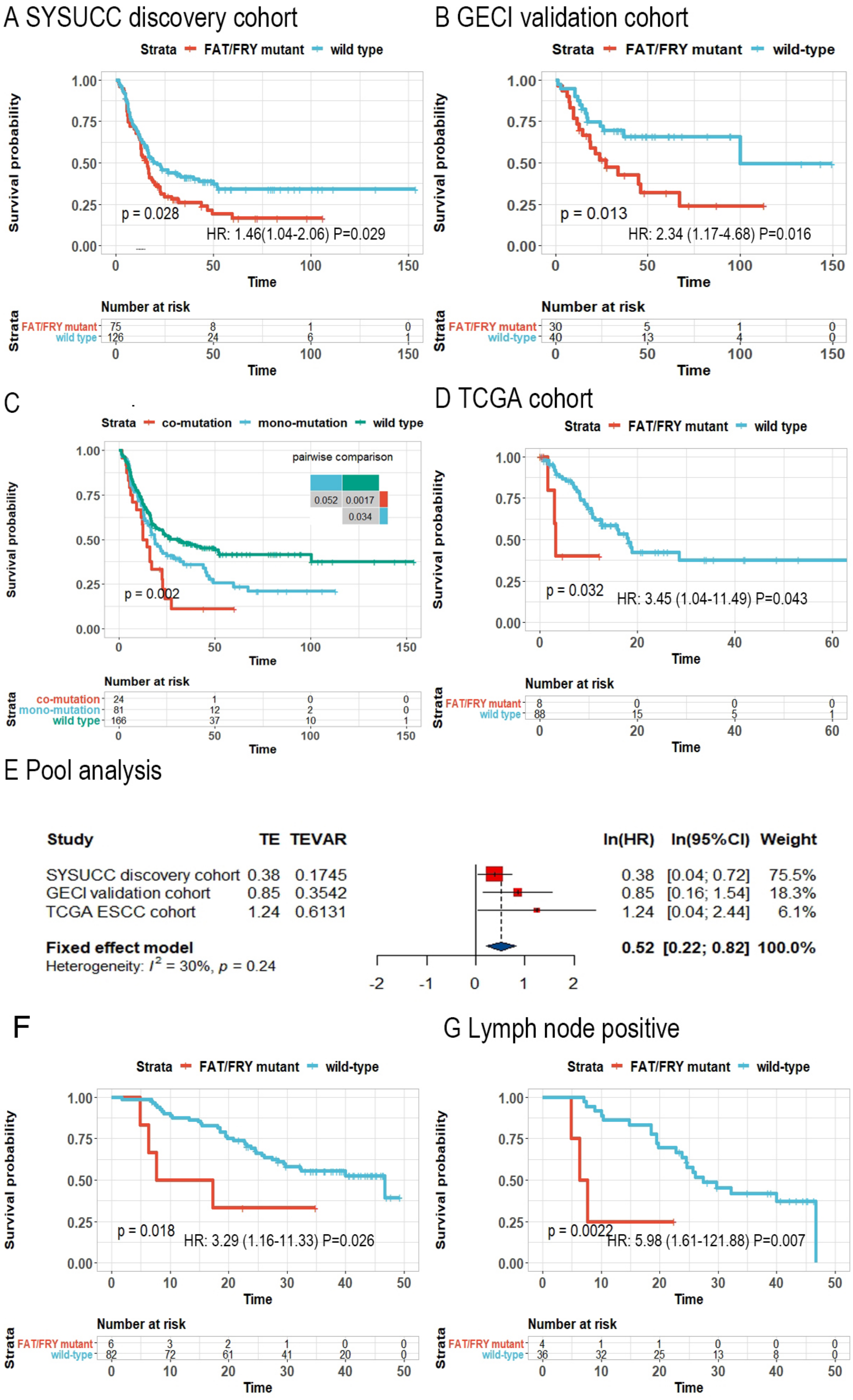
A SYSUCC discovery cohort
Strata FAT/FRY mutant wild type
Survival probability
p = 0.028
HR: 1.46(1.04-2.06) P=0.029
Time
Number at risk
FAT/FRY mutant 75 8 1 0
wild type 126 24 6 1
B GECI validation cohort
Strata FAT/FRY mutant wild-type
p = 0.013
HR: 2.34 (1.17-4.68) P=0.016
FAT/FRY mutant 30 5 1 0
wild-type 40 13 4 0
C
Strata co-mutation mono-mutation wild type
pairwise comparison
0.052 0.0017
0.034
p = 0.002
co-mutation 24 1 0 0
mono-mutation 81 12 2 0
wild type 166 37 10 1
D TCGA cohort
Strata FAT/FRY mutant wild type
p = 0.032
HR: 3.45 (1.04-11.49) P=0.043
FAT/FRY mutant 8 0 0 0
wild type 88 15 5 1
E Pool analysis
Study TE TEVAR ln(HR) ln(95%CI) Weight
SYSUCC discovery cohort 0.38 0.1745 0.38 [0.04; 0.72] 75.5%
GECI validation cohort 0.85 0.3542 0.85 [0.16; 1.54] 18.3%
TCGA ESCC cohort 1.24 0.6131 1.24 [0.04; 2.44] 6.1%
Fixed effect model 0.52 [0.22; 0.82] 100.0%
Heterogeneity: I^2 = 30%, p = 0.24
F
Strata FAT/FRY mutant wild-type
p = 0.018
HR: 3.29 (1.16-11.33) P=0.026
FAT/FRY mutant 6 3 2 1 0 0
wild-type 82 72 61 41 20 0
G Lymph node positive
Strata FAT/FRY mutant wild-type
p = 0.0022
HR: 5.98 (1.61-121.88) P=0.007
FAT/FRY mutant 4 1 1 0 0 0
wild-type 36 32 25 13 8 0

Figure S5 Association of the three-gene signature with patient prognosis. (A) Kaplan-Meier survival analysis showed that patients with mutation(s) in at least one mutation in genes of this signature had significantly shorter DFS than those patients with wild type genotype. (B) The prognostic value was further validated in an independent validation cohort (B). (C) Survival curves showed a marginal trend that patients with two mutations in three-gene signature had worse DFS than patients with one mutations in three-signature. (D) The 'FAT/FRY' subgroup had shorter progression-free survival in TCGA cohort. (E) Multivariate cox regression analysis in TCGA/ICG cohort showed that three-gene signature is associated with poor survival independent TNM stage and demographic variables. In TCGA cohort, 95 patients with definitive pathological stage were included in this analysis. (F) Forest plot of hazard ratio (HR) of relapse for 367 ESCC patients in different molecular subgroups from three cohorts with the fixed effects model. (pooled HR: 1.70, 95%CI: 1.27-2.93, I^2=38%, P=0.2). TE: effect, TEVAR: standard error of the effect. (F/G) The negative association between the three-gene signature and the OS in whole patients (F) and patients with lymph node metastasis (G) in another independent dataset[6].

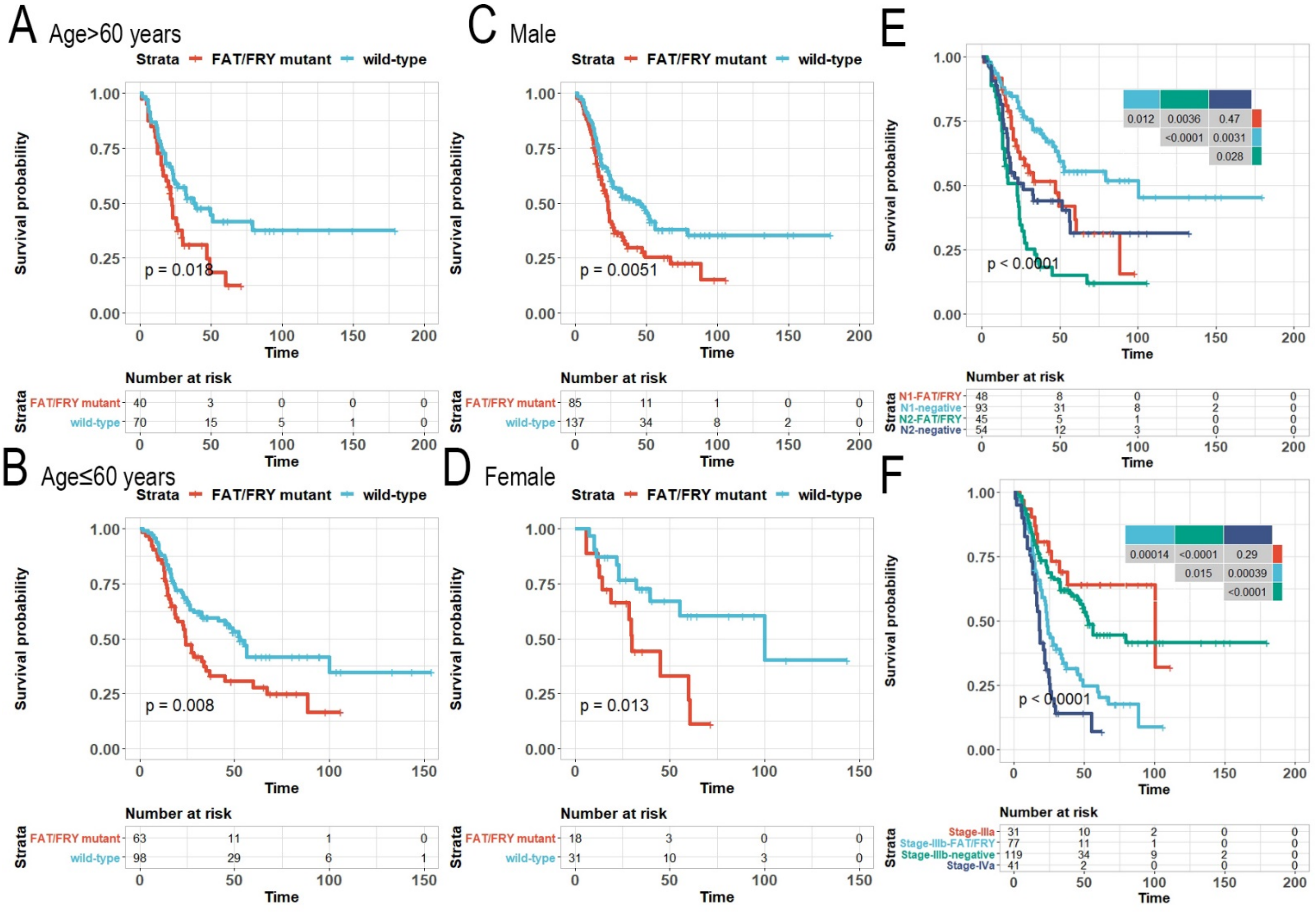

Figure S6 Survival curves of patients according to three-gene signature stratified by clinicopathological parameters. The three-gene mutation signature reflects poor prognosis in different demographic subgroups. OS curves of "FAT/FRY" mutant patients and wild type patients were stratified by gender (A/B), age (C/D), N stage (E) and AJCC 8th TNM stage (F). Patients in wild-type in stage IIIB had similar OS with patients in stage IIIA. Patients in wild-type group in N2 stage had similar OS with patients in FAT/FRY subtype in N1 stage.

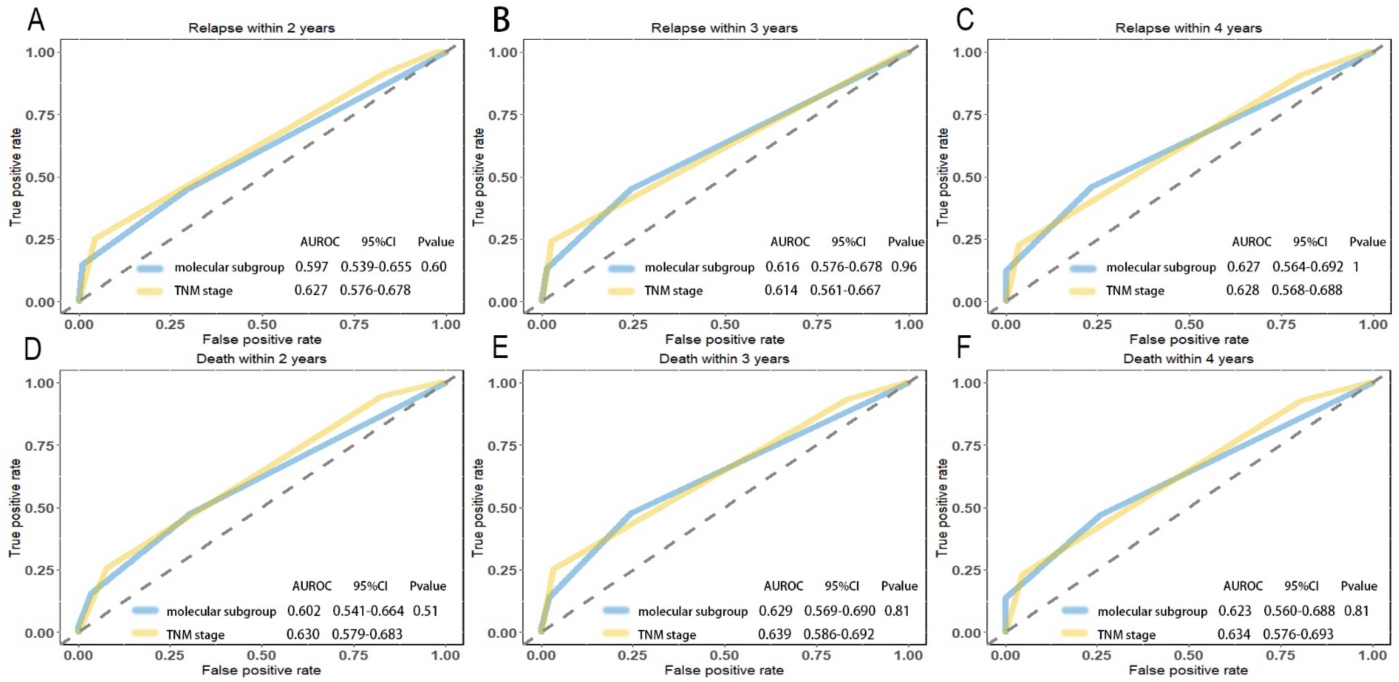

Figure S7 Comparisons of the sensitivity and specificity for prediction of disease progression and deathby the three-gene signature and the TNM stage. Receiver operating characteristics (ROC) curves for relapse within 2, 3 and 4 years (a-c), and disease progression within 2, 3 and 4 years (d-f). AUROC, area under the ROC (AUROC). Area under the curve was compared using t test.

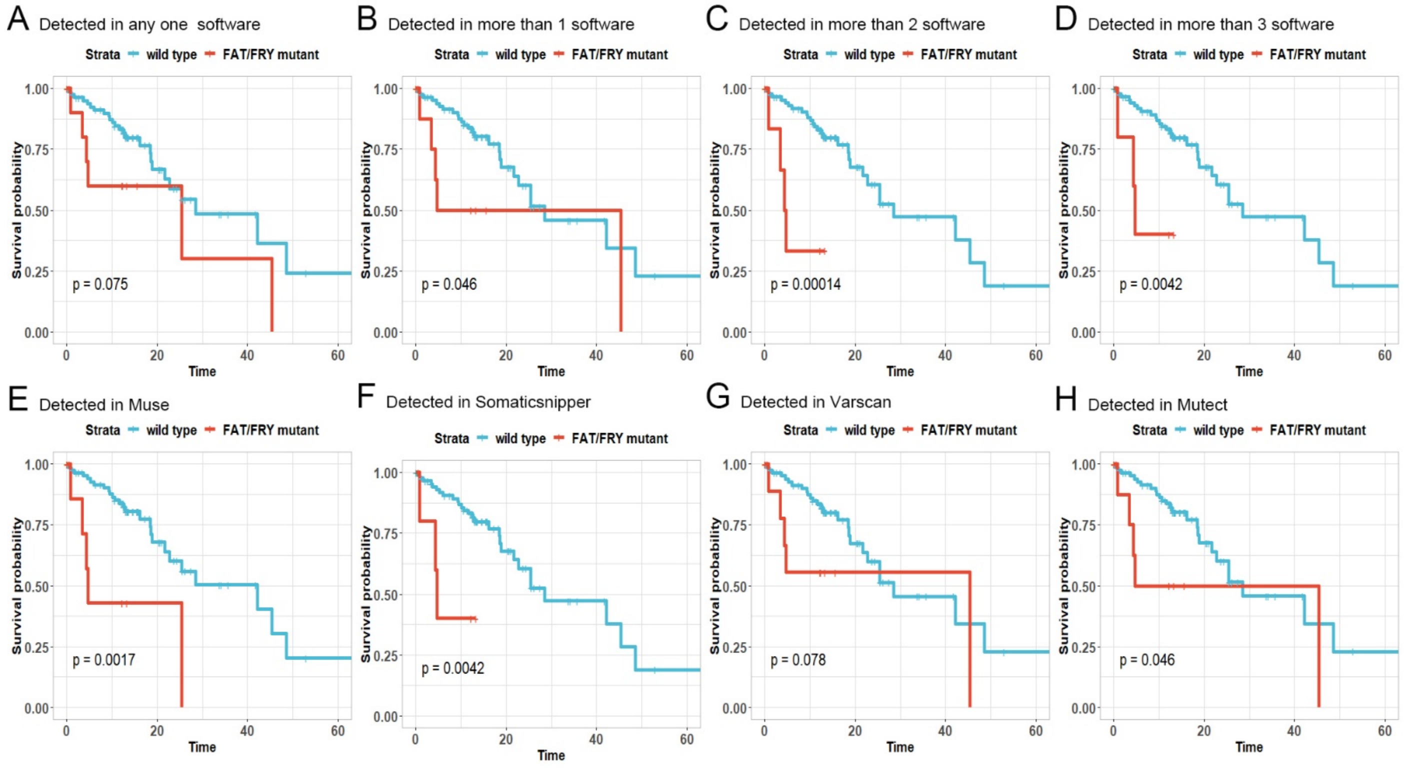

Figure S8 Prognostic value of the three-gene mutation signature detected by different methods in TCGA cohort. Patients were assigned into FAT/FRY subgroup according to their mutation status of FAT1, FAT3 and FRY.

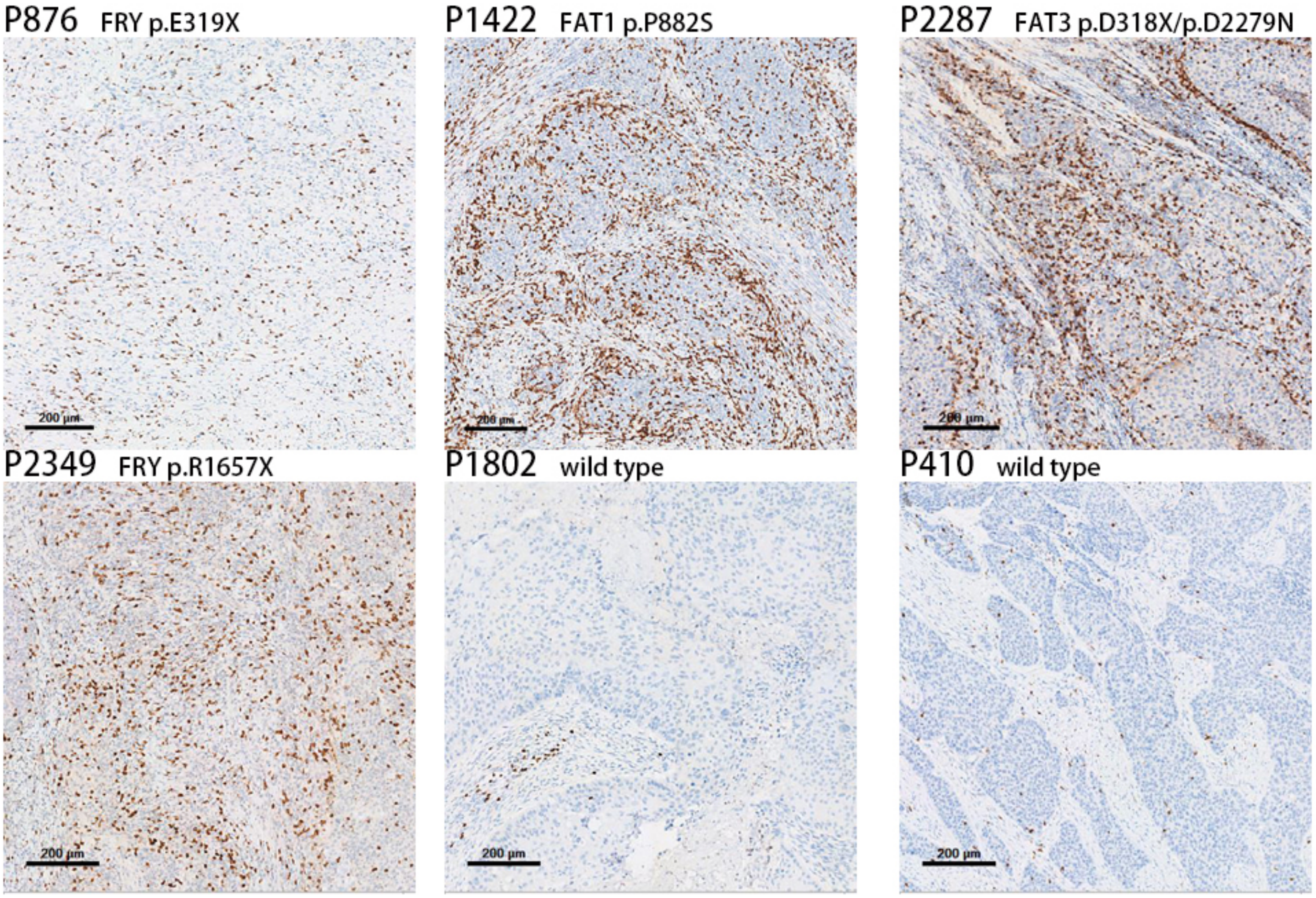

Figure S9 Representative images of CD8 immunohistochemistry of ESCC tissues from FAT/FRY muatant and wild type patients. The brown dot indicated the CD8 positive cell and the blue dot represented the nucleus.

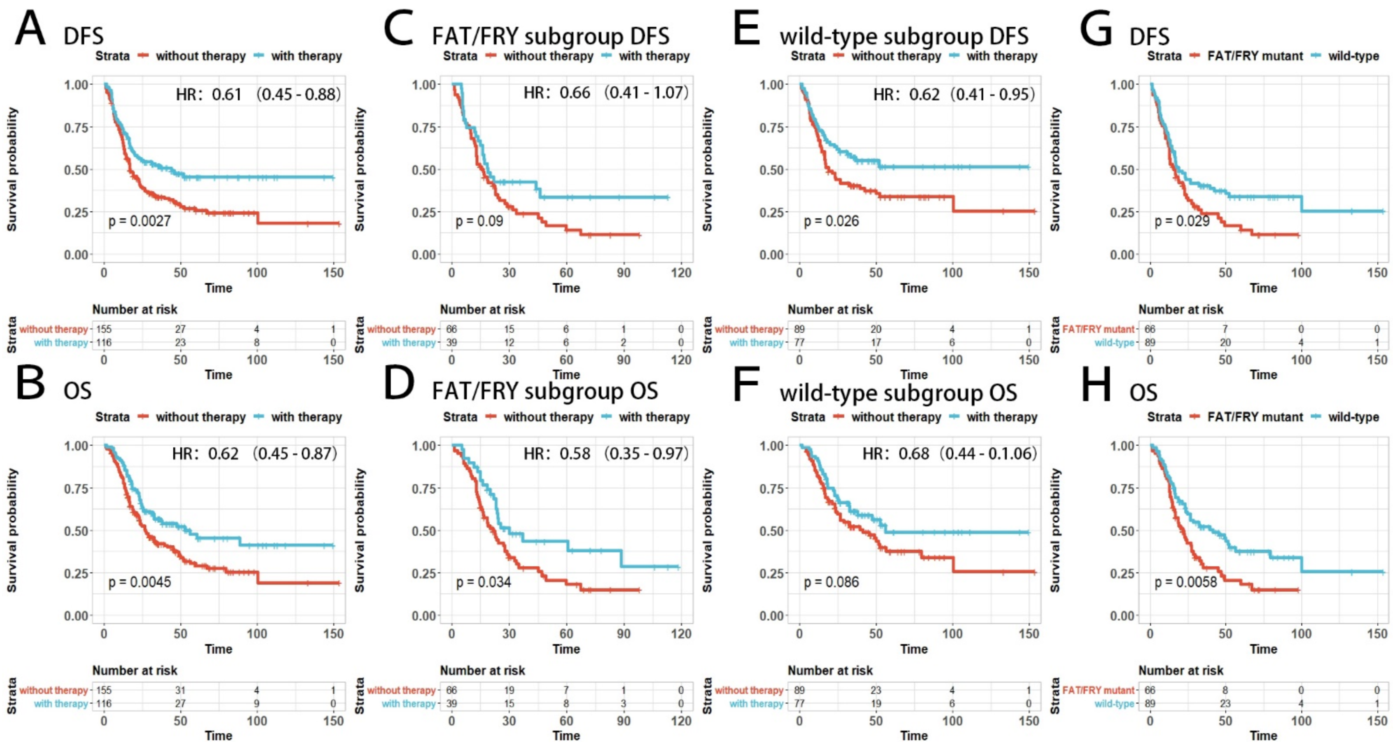

Figure S10 FAT/FRY signature is prognosticator instead of predictor of ajuvant

therapy. (A/B) Adjuvant therapy improved both DFS (A) and OS (B) in pN+ ESCC populations. (C-F) The effects of adjuvant therapy did not differed between the FAT/FRY subgroup (C/D) and wild-type subgroup (E-F). (G/H) In patients with surgery alone (without adjuvant therapy), FAT/FRY signature associated with poor DFS (G) and OS (H).

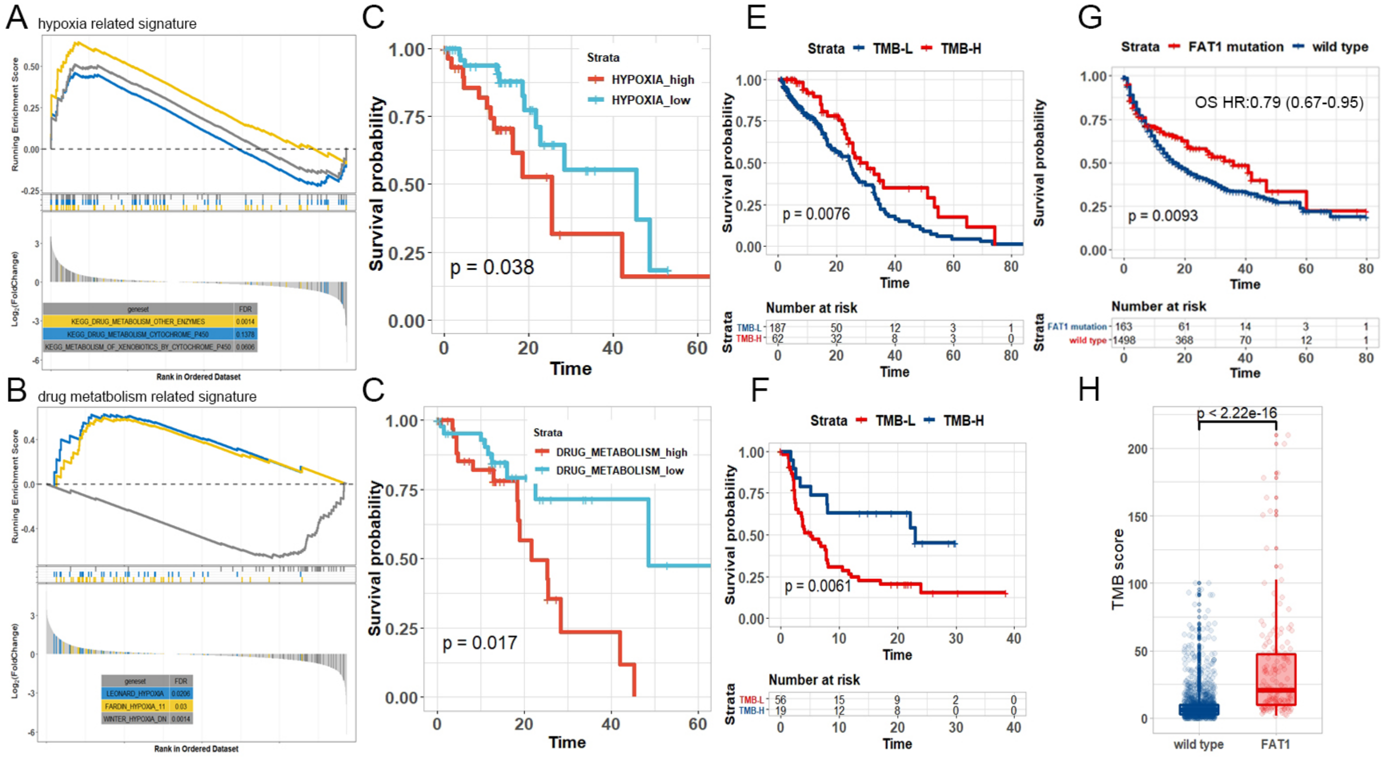

Figure S11 Prognostic value of genomic and transcriptomic features of FAT/FRY subgroup tumors.

(A/B) GSEA analysis revealed enrichment of hypoxia related genes (A) and drug-metabolism related genes (B) in FAT/FRY subtype ESCC. (C/D) Survival differences of patients subdivided by expression levels of hypoxia and drug metabolism associated signature. The red line represented patients with high score of the signature. (E/F) Prognosis differences of TMB-H and TMB-L patients in MSS tumor cohort (e) and NSCLC cohort (f). (G/H) As a core component of the FAT/FRY signature, FAT1 mutation was associated with longer OS and higher TMB in patients treated with ICIs in Samstein’s cohort.

Table S1 Detailed comparison of clinical variables between discovery cohort and independent validation cohort				
Variables	Discovery set(N=201) N(%)	Independent validation set(N=70) N(%)	P value	The entire set(N=271)
Sex			0.571	
Female	35(17.4)	15(21.4)		50(18.5)
Male	166(82.6)	55(88.6)		221(81.5)
Age			0.854	
＜59	96(47.8)	35(50)		130(48.0)
⩾60	105(52.2)	35(50)		141(52.0)
Smoking status			0.631	
Yes	129(64.2)	42(60.0)		171(63.1)
No	72(35.8)	28(40.0)		100(36.9)
Alcoholism			0.107	
Yes	99(49.3)	26 (37.1)		125(46.1)
No	102(50.7)	44(62.9)		146(53.9)
Differentiation			0.176	
Well	25(12.4)	14(20.0)		39(14.4)
Moderate-poor	176(87.4)	56(80.0)		232(85.6)
Surgical approach			0.77	
Left thoracotomy	66(32.8)	25(35.7)		91(33.6)
Right thoracotomy	135(67.2)	45(64.3)		180(66.4)
Lesion location			0.446	
Upper	16(8.0)	3(4.3)		19(7.4)
Middle	115(57.2)	45(64.3)		160(59.0)
Lower	70(34.8)	22(31.4)		92(35.1)
pT classification			0.669	
T1-T2	31(15.4)	13(18.6)		44(16.2)
T3-T4a	170(84.6)	57(81.4)		227(83.8)
pN classification			0.158	
N1	99(49.3)	42(60.0)		141(52.0)
N2-3	102(50.7)	28(40.0)		130(48.0)
Adjuvant chemo ± radiotherapy				
Yes	85(42.3)	31(44.3)	0.88	116(42.8)
No	116(57.7)	39(55.7)		155(57.2)

Table S1 Detailed comparison of clinical variables between discovery cohort and independent validation cohort

Table S5 Multivariable Cox regression analysis of the three-gene signature and clinicopathological factors				
Variable	Discovery cohort (N=201)		Validation cohort (N=70)	
	HR (95% CI)	P	HR (95% CI)	P
Sex (male vs female)	0.60 (0.36-0.99)	0.047	0.39 (0.14-1.09)	0.07
Age (≤60 vs >60)	0.93 (0.65-1.33)	0.68	0.73 (0.23-2.33)	0.60
pT classification pT_{3-4} VS pT_{1-2}	1.27 (0.75-2.17)	0.54	3.07 (1.04-10.45)	0.045
pN classification pN_{2-3} VS pN_{1}	3.22 (1.97-5.28)	<0.001	2.20 (0.61-4.09)	0.14
Differentiation pG_{3} VS pG_{1-2}	0.85 (0.51-1.43)	0.54	1.58 (0.61-4.09)	0.35
Number of lymph nodes examined (≤27 vs >27)	1.31 (0.92-1.88)	0.14	1.63 (0.78-3.37)	0.20
Prognostic subtype (FAT/FRY vs wild type)	1.47 (1.04-2.09)	0.028	2.82 (1.28-6.22)	0.01
Adjuvant chemo ± radiotherapy (Yes vs No)	0.67 (0.46-0.97)	0.035	0.33 (0.15-0.72)	0.006

Table S5 Multivariable Cox regression analysis of the three-gene signature and clinicopathological factors

Table S6 Detailed comparison of clinical variables between "FAT/FRY" group and wild type group across the 271 ESCC patients

Variables	FAT/FRY (N=105)	wild-type (N=166)	P	Overall (N=271)
Age			0.85	
≤60	53 (50.4)	87 (52.4)		140 (51.6)
＞60	52 (49.6)	79 (47.6)		131 (48.4)
Sex			0.97	
Female	19 (18.5)	31 (18.7)		50 (18.5)
Male	86 (71.5)	135 (71.3)		221 (81.5)
T stage			0.85	
T1	7 (6.7)	12 (7.7)		19 (7.0)
T2	32 (30.5)	48 (28.9)		80 (29.5)
T3	57 (54.3)	96 (57.8)		153 (56.5)
T4	9 (8.6)	10 (6.0)		19 (7.0)
N stage			0.16	
N1	48 (45.7)	91 (54.8)		139 (51.3)
N2	47 (44.8)	55 (33.1)		102 (37.6)
N3	10 (9.5)	20 (12.0)		30 (11.1)
Surgical procedure			0.93	
Right thoracotomy	70 (68.0)	112 (66.7)		180 (65.7)
Left thoracotomy	33 (32.0)	56 (33.3)		91 (34.3)
Differentiation			0.84	
G2-3	87 (82.9)	145 (87.3)		232 (85.6)
G1	18 (17.1)	21 (12.7)		39 (14.4)
smoking status			1	
Yes	66 (62.8)	105 (63.3)		171 (63.1)
No	39 (37.1)	61 (36.7)		100 (36.9)
alcohoism = 1 (%)			0.31	
Yes	53 (50.5)	72 (43.4)		125 (46.1)
No	52 (49.5)	94 (56.6)		146 (53.9)
position (%)			0.10	
Upper	3 (2.9)	16 (9.6)		19 (7.4)
Middle	66 (62.9)	94 (56.6)		160 (59.0)
Lower	36 (34.3)	56 (33.7)		92 (33.9)
Adjuvant therapy			0.16	
Yes	39 (37.1)	77 (46.4)		116 (42.8)
No	66 （62.9)	89 (53.6)		155 (57.2)

Table S6 Detailed comparison of clinical variables between "FAT/FRY" group and wild type group across the 271 ESCC patients

Signature Name	Resource	Supplmentary reference
CD 8 T cells	Charoentong et al. Cell Rep 2017;18:248-262.	[41]
Eosinophils	Charoentong et al. Cell Rep 2017;18:248-262.	[41]
Gamma delta T cells	Charoentong et al. Cell Rep 2017;18:248-262.	[41]
Activated dendritic cells	Charoentong et al. Cell Rep 2017;18:248-262.	[41]
IFN gamma signature	Ayers M, et al. J Clin Invest 2017;127:2930-2940	[53]
GO BP Hippo signaling	MsigDB	http://www.gsea-msigdb.org/
LEONARD_HYPOXIA	MsigDB	http://www.gsea-msigdb.org/
LEONARD_HYPOXIA	MsigDB	http://www.gsea-msigdb.org/
FARDIN_HYPOXIA	MsigDB	http://www.gsea-msigdb.org/
WINTER_HYPOXIA_DN	MsigDB	http://www.gsea-msigdb.org/
KEGG_DRUG_METABOLISM_OTHER_ENZYMES	MsigDB	http://www.gsea-msigdb.org/
KEGG_DRUG_METABOLISM_CYP450	MsigDB	http://www.gsea-msigdb.org/
KEGG_DRUG_XENOBIOTICS_CYP450	MsigDB	http://www.gsea-msigdb.org/

Table S8 Publicly-available gene signatures used in the study

CTGF former	CTCGCGGCTTACCGACTG
CTGF reverse	GGCTCTGCTTCTCTAGCCTG
CYR61 former	GTTTGGCCCAGACCCAACTA
CYR61 reverse	GGCTCTGCTTCTCTAGCCTG
LATS2 former	CAGGATGCGACCAGGAGATG
LATS2 reverse	CAGGATGCGACCAGGAGATG
FAT1 former	TTCAAAATAGGTGAAGAGACAGGTG
FAT1 reverse	TTCAAAATAGGTGAAGAGACAGGTG

Table S11 Primers used in our qRT-PCR experiment.

1. Liu J, Lichtenberg T, Hoadley KA et al. An Integrated TCGA Pan-Cancer Clinical Data Resource to Drive High-Quality Survival Outcome Analytics, Cell 2018;173:400-416 e411.
2. Dai W, Ko JMY, Choi SSA et al. Whole-exome sequencing reveals critical genes underlying metastasis in oesophageal squamous cell carcinoma, J Pathol 2017;242:500-510.
3. Sawada G, Niida A, Uchi R et al. Genomic Landscape of Esophageal Squamous Cell Carcinoma in a Japanese Population, Gastroenterology 2016;150:1171-1182.

4. Qin HD, Liao XY, Chen YB et al. Genomic Characterization of Esophageal Squamous Cell Carcinoma Reveals Critical Genes Underlying Tumorigenesis and Poor Prognosis, Am J Hum Genet 2016;98:709-727.
5. Zhang L, Zhou Y, Cheng C et al. Genomic analyses reveal mutational signatures and frequently altered genes in esophageal squamous cell carcinoma, Am J Hum Genet 2015;96:597-611.
6. Song Y, Li L, Ou Y et al. Identification of genomic alterations in oesophageal squamous cell cancer, Nature 2014;509:91-95.
7. Lin DC, Hao JJ, Nagata Y et al. Genomic and molecular characterization of esophageal squamous cell carcinoma, Nat Genet 2014;46:467-473.
8. Gao YB, Chen ZL, Li JG et al. Genetic landscape of esophageal squamous cell carcinoma, Nat Genet 2014;46:1097-1102.
9. Agrawal N, Jiao Y, Bettegowda C et al. Comparative genomic analysis of esophageal adenocarcinoma and squamous cell carcinoma, Cancer Discov 2012;2:899-905.
10. Li H, Durbin R. Fast and accurate short read alignment with Burrows-Wheeler transform, Bioinformatics 2009;25:1754-1760.
11. Faust GG, Hall IM. SAMBLASTER: fast duplicate marking and structural variant read extraction, Bioinformatics 2014;30:2503-2505.
12. Cibulskis K, Lawrence MS, Carter SL et al. Sensitive detection of somatic point mutations in impure and heterogeneous cancer samples, Nat Biotechnol 2013;31:213-219.
13. Wang K, Li M, Hakonarson H. ANNOVAR: functional annotation of genetic variants from high-throughput sequencing data, Nucleic Acids Res 2010;38:e164.
14. Forbes SA, Bindal N, Bamford S et al. COSMIC: mining complete cancer genomes in the Catalogue of Somatic Mutations in Cancer, Nucleic Acids Res 2011;39:D945-950.
15. Lek M, Karczewski KJ, Minikel EV et al. Analysis of protein-coding genetic variation in 60,706 humans, Nature 2016;536:285-291.
16. Tamborero D, Gonzalez-Perez A, Lopez-Bigas N. OncodriveCLUST: exploiting the positional clustering of somatic mutations to identify cancer genes, Bioinformatics 2013;29:2238-2244.
17. Vaser R, Adusumalli S, Leng SN et al. SIFT missense predictions for genomes, Nat Protoc 2016;11:1-9.
18. Wagner A. Rapid detection of positive selection in genes and genomes through variation clusters, Genetics 2007;176:2451-2463.
19. Chang MT, Asthana S, Gao SP et al. Identifying recurrent mutations in cancer reveals widespread lineage diversity and mutational specificity, Nat Biotechnol 2016;34:155-163.
20. Nadeu F, Delgado J, Royo C et al. Clinical impact of clonal and subclonal TP53, SF3B1, BIRC3, NOTCH1, and ATM mutations in chronic lymphocytic leukemia, Blood 2016;127:2122-2130.
21. Rossi D, Khiabanian H, Spina V et al. Clinical impact of small TP53 mutated subclones in chronic lymphocytic leukemia, Blood 2014;123:2139-2147.
22. Talevich E, Shain AH, Botton T et al. CNVkit: Genome-Wide Copy Number Detection and Visualization from Targeted DNA Sequencing, PLoS Comput Biol 2016;12:e1004873.
23. Papaemmanuil E, Gerstung M, Malcovati L et al. Clinical and biological implications of driver mutations in myelodysplastic syndromes, Blood 2013;122:3616-3627; quiz 3699.
24. Papaemmanuil E, Gerstung M, Bullinger L et al. Genomic Classification and Prognosis in Acute Myeloid Leukemia, N Engl J Med 2016;374:2209-2221.
25. Yan T, Cui H, Zhou Y et al. Multi-region sequencing unveils novel actionable targets and spatial heterogeneity in esophageal squamous cell carcinoma, Nat Commun 2019;10:1670.
26. Sondka Z, Bamford S, Cole CG et al. The COSMIC Cancer Gene Census: describing genetic dysfunction across all human cancers, Nat Rev Cancer 2018;18:696-705.
27. Vogelstein B, Papadopoulos N, Velculescu VE et al. Cancer genome landscapes, Science 2013;339:1546-1558.
28. Kandoth C, McLellan MD, Vandin F et al. Mutational landscape and significance across 12 major cancer types, Nature 2013;502:333-339.
29. Tamborero D, Gonzalez-Perez A, Perez-Llamas C et al. Comprehensive identification of mutational cancer driver genes

across 12 tumor types, Sci Rep 2013;3:2650.

30. Patel BJ, Przychodzen B, Thota S et al. Genomic determinants of chronic myelomonocytic leukemia, Leukemia 2017;31:2815-2823.

31. Nik-Zainal S, Van Loo P, Wedge DC et al. The life history of 21 breast cancers, Cell 2012;149:994-1007.

32. Augustin T. Bradley-Terry-Luce models to incorporate within-pair order effects: representation and uniqueness theorems, Br J Math Stat Psychol 2004;57:281-294.

33. Landau DA, Carter SL, Stojanov P et al. Evolution and impact of subclonal mutations in chronic lymphocytic leukemia, Cell 2013;152:714-726.

34. Landau DA, Tausch E, Taylor-Weiner AN et al. Mutations driving CLL and their evolution in progression and relapse, Nature 2015;526:525-530.

35. Dentr SC. Principles of reconstructing the subclonal architecture of cancers.

36. Brunet J-P. Metagenes and molecular pattern discovery using matrix factorization, pnas 2004.

37. Wilkerson MD, Hayes DN. ConsensusClusterPlus: a class discovery tool with confidence assessments and item tracking, Bioinformatics 2010;26:1572-1573.

38. Paola Sebastiani ISK, Marco F. Ramoni. Consensus Clustering: A Resampling-Based Method for Class Discovery and Visualization of Gene Expression Microarray Data 2003.

39. Cancer Genome Atlas Research N. Integrated genomic characterization of oesophageal carcinoma, Nature 2017;541:169-175.

40. Love MI, Huber W, Anders S. Moderated estimation of fold change and dispersion for RNA-seq data with DESeq2, Genome Biol 2014;15:550.

41. Charoentong P, Finotello F, Angelova M et al. Pan-cancer Immunogenomic Analyses Reveal Genotype-Immunophenotype Relationships and Predictors of Response to Checkpoint Blockade, Cell Rep 2017;18:248-262.

42. Lee E, Chuang HY, Kim JW et al. Inferring pathway activity toward precise disease classification, PLoS Comput Biol 2008;4:e1000217.

43. Bindea G, Mlecnik B, Tosolini M et al. Spatiotemporal dynamics of intratumoral immune cells reveal the immune landscape in human cancer, Immunity 2013;39:782-795.

44. Chen PL, Roh W, Reuben A et al. Analysis of Immune Signatures in Longitudinal Tumor Samples Yields Insight into Biomarkers of Response and Mechanisms of Resistance to Immune Checkpoint Blockade, Cancer Discov 2016;6:827-837.

45. Hugo W, Zaretsky JM, Sun L et al. Genomic and Transcriptomic Features of Response to Anti-PD-1 Therapy in Metastatic Melanoma, Cell 2016;165:35-44.

46. Snyder A, Nathanson T, Funt SA et al. Contribution of systemic and somatic factors to clinical response and resistance to PD-L1 blockade in urothelial cancer: An exploratory multi-omic analysis, PLoS Med 2017;14:e1002309.

47. Samstein RM, Lee CH, Shoushtari AN et al. Tumor mutational load predicts survival after immunotherapy across multiple cancer types, Nat Genet 2019;51:202-206.

48. Miao D, Margolis CA, Vokes NI et al. Genomic correlates of response to immune checkpoint blockade in microsatellite-stable solid tumors, Nat Genet 2018;50:1271-1281.

49. Hellmann MD, Nathanson T, Rizvi H et al. Genomic Features of Response to Combination Immunotherapy in Patients with Advanced Non-Small-Cell Lung Cancer, Cancer Cell 2018;33:843-852 e844.

50. Cui Y, Chen H, Xi R et al. Whole-genome sequencing of 508 patients identifies key molecular features associated with poor prognosis in esophageal squamous cell carcinoma, Cell Res 2020.

51. Cancer Genome Atlas Research N, Analysis Working Group: Asan U, Agency BCC et al. Integrated genomic characterization of oesophageal carcinoma, Nature 2017;541:169-175.

52. Yang W, Soares J, Greninger P et al. Genomics of Drug Sensitivity in Cancer (GDSC): a resource for therapeutic biomarker discovery in cancer cells, Nucleic Acids Res 2013;41:D955-961.

53. Ayers M, Lunceford J, Nebozhyn M et al. IFN-gamma-related mRNA profile predicts clinical response to PD-1 blockade, J Clin Invest 2017;127:2930-2940.
